# Mapping and Characterization of a Wheat Stem Rust Resistance Gene in Durum Wheat “Kronos”

**DOI:** 10.3389/fpls.2021.751398

**Published:** 2021-10-15

**Authors:** Hongna Li, Lei Hua, Matthew N. Rouse, Tianya Li, Shuyong Pang, Shengsheng Bai, Tao Shen, Jing Luo, Hongyu Li, Wenjun Zhang, Xiaodong Wang, Jorge Dubcovsky, Shisheng Chen

**Affiliations:** ^1^Peking University Institute of Advanced Agricultural Sciences, Weifang, China; ^2^Department of Plant Sciences, University of California, Davis, Davis, CA, United States; ^3^College of Plant Protection, Shenyang Agricultural University, Shenyang, China; ^4^US Department of Agriculture-Agricultural Research Service, Cereal Disease Laboratory and Department of Plant Pathology, University of Minnesota, St. Paul, MN, United States; ^5^Howard Hughes Medical Institute, Chevy Chase, MD, United States; ^6^State Key Laboratory of North China Crop Improvement and Regulation, College of Plant Protection, Hebei Agricultural University, Baoding, China

**Keywords:** durum wheat, stem rust, resistance gene, *SrKN*, introgression

## Abstract

Wheat stem (or black) rust is one of the most devastating fungal diseases, threatening global wheat production. Identification, mapping, and deployment of effective resistance genes are critical to addressing this challenge. In this study, we mapped and characterized one stem rust resistance (*Sr*) gene from the tetraploid durum wheat variety Kronos (temporary designation *SrKN*). This gene was mapped on the long arm of chromosome 2B and confers resistance to multiple virulent *Pgt* races, such as TRTTF and BCCBC. Using a large mapping population (3,366 gametes), we mapped *SrKN* within a 0.29 cM region flanked by the sequenced-based markers *pku4856F2R2* and *pku4917F3R3*, which corresponds to 5.6- and 7.2-Mb regions in the Svevo and Chinese Spring reference genomes, respectively. Both regions include a cluster of nucleotide binding leucine-repeat (NLR) genes that likely includes the candidate gene. An allelism test failed to detect recombination between *SrKN* and the previously mapped *Sr9e* gene. This result, together with the similar seedling resistance responses and resistance profiles, suggested that *SrKN* and *Sr9e* may represent the same gene. We introgressed *SrKN* into common wheat and developed completely linked markers to accelerate its deployment in the wheat breeding programs. *SrKN* can be a valuable component of transgenic cassettes or gene pyramids that includes multiple resistance genes to control this devastating disease.

## Introduction

The total world human population is expected to increase 35% by 2050, which will require an increase of current food production levels by 70–100% (Godfray et al., 2010). Wheat, *Triticum aestivum* L. (*2n* = *6x* = 42, AABBDD) and *Triticum turgidum* subsp. *durum* (Desf.) Husn. (*2n* = *4x* = 28, AABB), provide roughly 20% of calories consumed by the human population and play a major role in global food security. To achieve further increases in wheat production, it is critical to reduce yield losses caused by the fungal pathogens. *Puccinia graminis* f. sp. *tritici* (*Pgt*), the causal agent of wheat stem (or black) rust, is one of the most yield-limiting diseases throughout the wheat-growing regions worldwide (Leonard, 2001). For the past several decades, stem rust has been effectively controlled by the use of genetic resistance and eliminating the alternate host barberry (*Berberis vulgaris* L.) (Peterson et al., 2005; Singh et al., 2015).

Unfortunately, this disease reemerged as a serious threat with the detection of a highly virulent isolate TTKSK (also known as Ug99) in Uganda in 1998. Ug99 is virulent to most of the deployed stem rust resistance genes, such as the widely deployed *Sr31* gene (Pretorius et al., 2000; Jin et al., 2007). Currently, 13 variants in the Ug99 lineage have been detected in the 13 countries extending from Africa to Asia (Nazari et al., 2009; Bhardwaj et al., 2019). Additional challenges are emerging from the appearance of other virulent races unrelated to the Ug99 race group, such as TRTTF, JRCQC, TKTTF, and TTRTF (Olivera et al., 2012, 2015; Tesfaye et al., 2020).

The non-Ug99 race TRTTF, which was first discovered in Yemen and subsequently in East Africa, defeated the resistance conferred by genes *SrTmp, Sr36*, and *Sr1RS*^*Amigo*^ that are effective against Ug99 (Olivera et al., 2012). The races TRTTF and JRCQC overcame the resistance provided by genes *Sr9e* and *Sr13* (Olivera et al., 2012), which are important sources of stem rust resistance in many commercial durum wheat cultivars (Periyannan et al., 2014; Singh et al., 2015). Virulent race TKTTF was responsible for a severe stem rust epidemic in the south of Ethiopia and caused nearly 100% yield losses on the Ug99 resistant wheat variety “Digalu” (Olivera et al., 2015). Another race of concern is TTRTF, which was first identified in Georgia in 2014 (Olivera et al., 2019), and subsequently spread to more countries, such as Hungary, Egypt, and Ethiopia (Tesfaye et al., 2020). Since *Pgt* has already demonstrated its ability for rapid spread and evolution, additional sources of resistance are needed to diversify the combinations of deployed *Sr* genes, including those from the primary wheat gene pool.

*Triticum turgidum* ssp. *durum*, which is part of the wheat primary gene pool, is grown in about 18 million ha worldwide with an annual production of approximately 35 million tons (Cakmak et al., 2010). Tetraploid wheat (*T. turgidum* ssp.) has contributed several stem rust resistance genes, including *Sr9d*/*Sr9e*/*Sr9g, Sr11, Sr12, Sr13a*/*Sr13b, Sr14*, and *Sr17* (Bariana, 2008; Singh et al., 2011, 2015; Zhang et al., 2017). The recent development of next-generation sequencing (NGS) and genome-wide high throughput genotyping platforms, such as the Illumina iSelect single nucleotide polymorphism (SNP) array (Illumina Inc., CA, USA) (Wang et al., 2014) and the wheat exome capture (Krasileva et al., 2017), have accelerated the identification of new stem rust resistance genes (Letta et al., 2014; Nirmala et al., 2017; Miedaner et al., 2019; Megerssa et al., 2020).

The durum wheat variety “Kronos” (PI 576168) developed by Arizona Plant Breeders Inc. (AZ, USA) was previously postulated to carry *Sr13* and a second TRTTF resistance gene, temporarily designated as *SrKN* (Zhang et al., 2017). *Sr13* has been cloned and encodes a typical coiled-coil nucleotide-binding leucine-rich repeat protein (Zhang et al., 2017). The objectives of this study were to: (1) characterize and genetically map *SrKN*; (2) identify the corresponding regions in the different sequenced wheat genomes; and (3) introgress the chromosome segment carrying *SrKN* into hexaploid wheat.

## Materials and Methods

### Plant Materials and Mapping Population

To map the TRTTF resistance gene, the Kronos *sr13* mutant line T4-3102, carrying a premature stop codon in the LRR domain, was crossed with the susceptible durum line Rusty (Klindworth et al., 2006). For the initial map, we evaluated a subset of 90 F_2_ plants with *Pgt* race TRTTF (isolate 06YEM34-1) and a separate subset of 145 F_2_ plants from the same population with BCCBC (isolate 09CA115-2). We tested the observed segregation ratios using χ^2^ tests.

For the construction of the high-resolution genetic map, we selected four F_2_ plants (plants 17, 31, 47, and 87) heterozygous for the *SrKN* candidate region using molecular markers and produced 1,468 F_3_ plants. These plants were genotyped with *SrKN* flanking markers to identify recombination events in the candidate gene region. The plants carrying these recombination events and their F_4_ progenies were challenged with *Pgt* races BCCBC and 34MKGQM.

To evaluate the resistance profile of *SrKN* to multiple *Pgt* races, we developed a pair of F_5_ sister lines homozygous for the presence (Td31-5R) or absence (Td31-7S) of *SrKN* using molecular markers and their levels of resistance to race BCCBC. This additional criterion was used to eliminate a minor *Sr* resistance gene present in T4-3102 that confers a mild resistance to BCCBC but not to TRTTF (as shown in the Results section). Td31-7S was F_4_ plant number 7 from F_3_ family 31, which was very susceptible to BCCBC. Td31-5R was F_4_ plant number 5 from the same segregating family, which carried the *SrKN* based on the flanking markers, but that showed an intermediate resistance reaction to BCCBC (we assumed that the very resistant parental line T4-3102 carries both genes).

Finally, we used a collection of 23 accessions of *T. turgidum* ssp. *durum* and 16 of *T. aestivum* to determine the value of the closely linked markers identified in this study for marker-assisted selection.

### Stem Rust Assays

The infection types (IT) of mutant line T4-3102 and Rusty to *Pgt* races TTKSK (isolate 04KEN156/04), TRTTF (06YEM34-1), TKTTF (13ETH18-1), and JRCQC (09ETH08-3) were reported in the previous study (Zhang et al., 2017). In this study, the parental lines T4-3102 and Rusty, and their segregating populations were re-evaluated with races TRTTF (06YEM34-1) and BCCBC (09CA115-2) at the United States Department of Agriculture-Agricultural Research Service (USDA-ARS) and Cereal Disease Laboratory and the University of California, Davis (UCD), respectively. Evaluations with four Chinese *Pgt* races 21C3CTTTM (20GH13), 34MKGQM (20IAL06), 34MTGSM (20GSA1), and 34C3RTGQM (20IAL32) were performed at Peking University Institute of Advanced Agricultural Sciences, Weifang, Shandong, China.

The avirulence/virulence formulae of the *Pgt* races used in this study are presented in Supplementary Table S1. The procedures for inoculation were as described previously (Rouse et al., 2011) and ITs were scored using a 0–4 scale also described before (Stakman et al., 1962; Rouse et al., 2011; Chen et al., 2015). The additional symbols “+” or “–” were used to indicate larger or smaller pustules within the same IT (Roelfs and Martens, 1988).

### Wheat 90K iSelect Assay

Genomic DNA of the parents and F_2_ plants was extracted using the cetyltrimethylammonium bromide (CTAB) method (Murray and Thompson, 1980). The quality and quantity of DNA were measured using a NanoDrop Spectrophotometer (Thermo Fisher Scientific, MA, USA) and normalized to 50 ng/μl. We genotyped the parental lines and 46 F_2_ plants at the USDA-ARS Small Grain Genotyping Lab at Fargo (ND, USA) with the wheat 90K SNP iSelect Illumina platform (Wang et al., 2014). The SNP genotype calling was processed using Illumina GenomeStudio v.2011.1 (Illumina Inc., CA, USA). The polymorphic SNP markers with more than 20% missing values were removed.

### Marker Development

Once the linked SNPs were identified using the 90K SNP array, their flanking sequences were used to perform BLASTN (Basic Local Alignment Search Tool for nucleotide sequence) searches in the reference genomes of hexaploid wheat Chinese Spring (CS) (The International Wheat Genome Sequencing Consortium, 2018) and tetraploid wheat Svevo (Maccaferri et al., 2019) to define the *SrKN* candidate region in these two genomes. To accelerate the development of markers in the candidate region, we performed exome-capture for the susceptible parent Rusty (accession number PRJNA751176), since the sequence of Kronos assembly (Walkowiak et al., 2020) was already available. Genomic library preparation, exome capture, sequencing, and data analysis were conducted using the same methods as described before (Krasileva et al., 2017; Mo et al., 2018). We aligned the Rusty and Kronos sequences of the genes in the candidate region, identified the polymorphic sites, and generated sequence-based markers spaced throughout the candidate gene region.

DNA amplification was carried out in a Veriti 96-Well Fast Thermal Cycler with the following thermal cycling profile: an initial denaturation step of 94°C for 3 min, followed by 35 cycles consisting of 94°C for 30 s, annealing at 50–65°C for 30 s, and extension at 72°C for 60 s, ending with a final step at 72°C for 10 min. After the PCR amplification, 10 μl PCR products were subjected to agarose gel electrophoresis (~1.5% agarose), and the gels were stained with ethidium bromide.

### Allelism Test

The tetraploid durum wheat variety Vernal was originally hypothesized to have both *Sr9e* and *Sr13* (Saini et al., 2018). However, using a published diagnostic marker for *Sr13* (Zhang et al., 2017), we found that Vernal carries the *Sr13* susceptible haplotype (S7). To obtain the *Sr9e* monogenic line, Vernal was crossed with susceptible line Rusty, and the resulting F_1_ was backcrossed two times with Rusty. The presence of the Vernal allele in the *Sr9e* region was monitored during backcrossing using the cleaved amplified polymorphic sequence (CAPS) markers *pku4861F7R7* and *pku4922F1R2*. The *Sr9e* monogenic line (referred hereafter as Vernal*-*BF9e) was selected from the BC_2_F_2_ plants. An allelism test between *SrKN* and *Sr9e* was carried out using 470 F_2_ plants derived from the cross between the monogenic lines Td31-5R (*SrKN*) × Vernal*-*BF9e (*Sr9e*) inoculated with *Pgt* race 34MKGQM.

### Transferring of *T. durum* Segment Carrying *SrKN* Into Hexaploid Wheat

*Triticum turgidum* subsp. *durum* wheat variety Kronos was crossed with the *T. monococcum* wheat accession PI 306540 (A^m^A^m^) as described before (Chen et al., 2020). The resulting F_1_ triploid plants were completely male sterile and were crossed with common wheat variety Clear White (PVP 2004-00244). Next, the F_1_ plants were backcrossed to the hexaploid wheat line Fielder. Flanking and completely linked PCR markers (**Table 2**) were used to validate the presence of Kronos segment, including *SrKN* during backcrossing. One BC_2_F_2_ plant heterozygous for the *SrKN* candidate chromosome region and without other *Sr* resistance genes was self-pollinated. The selected BC_2_F_3_ plants were divided into two groups and inoculated with *Pgt* races 34MKGQM and 34C3RTGQM, respectively. After phenotyping, the BC_2_F_3_ plants homozygous for *SrKN* were transplanted and then, self-pollinated to generate the BC_2_F_4_ seeds.

### Statistical Analyses

We mapped the Rusty reads from the exome capture on the Kronos assembly and called SNPs using SAMtools. We generated the pileup files and used BCFtools for variant calling (http://samtools.sourceforge.net/). The variants with a sequencing depth of ≤ 5 and mapping quality of ≤ 50 were removed for subsequent analysis. The polymorphic markers and the stem rust resistance phenotypes were used to construct the genetic linkage maps using the software JoinMap 4.0 and MapChart 2.2 (Kyazma BV, Wageningen, Netherlands; https://www.wur.nl/en/show/Mapchart.htm) (Stam, 1993; Voorrips, 2002; Van Ooijen, 2006). The BLASTN searches against the hexaploid wheat CS (https://wheat-urgi.versailles.inra.fr/Seq-Repository/BLAST), and the tetraploid wheat Svevo and Kronos (https://wheat.pw.usda.gov/blast/) were used to assist the marker development.

## Results

### Characterization of Stem Rust Resistance in Durum Wheat Line T4-3102

**Figure 1:**
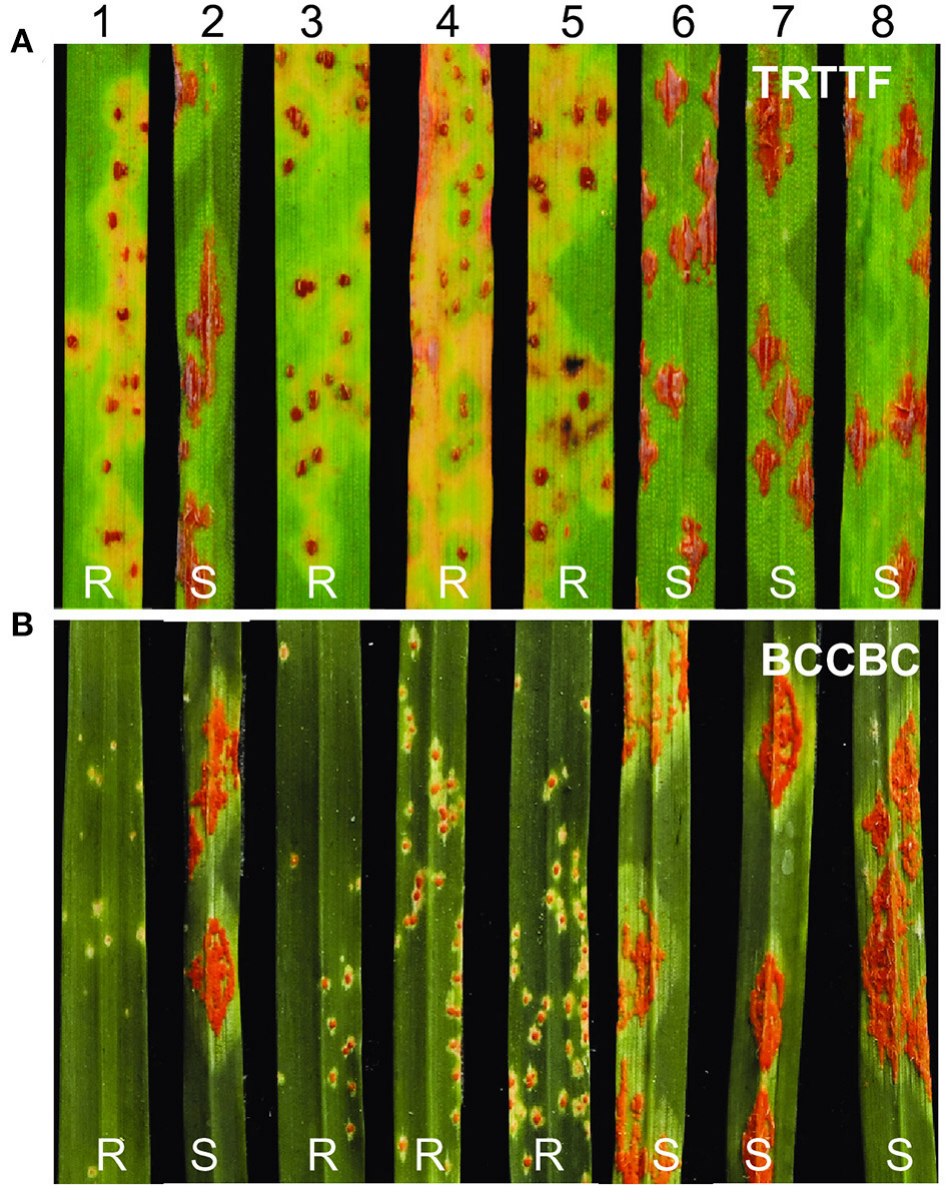
Reactions to *Pgt* races TRTTF and BCCBC in segregating population. **(A)** Inoculated with race TRTTF. **(B)** Inoculated with race BCCBC. 1, T4-3102 (*SrKN*); 2, Rusty; 3–5, resistant plants; 6–8, susceptible plants. R, resistant; S, susceptible.

In the seedling tests, the durum wheat line T4-3102 displayed resistant ITs (ITs = 1; to 1+) to *Pgt* race TRTTF (isolate 06YEM34-1), whereas Rusty exhibited ITs of “3+” to “4” (Figure 1A). In a subset of 90 F_2_ plants from the cross, T4-3102 × Rusty evaluated with TRTTF, the plants with ITs ranging between “1;” and “1+” (similar to T4-3102) were classified as resistant and those with ITs from “3+” to “4” (similar to Rusty) were recorded as susceptible (Figure 1A). Among them, 69 plants were resistant and 21 were susceptible, which fits well the 3:1 (resistant:susceptible) segregation ratio expected for a single genetic locus (χ^2^ = 0.13, *P* = 0.72).

In seedling of the two parental lines inoculated with race BCCBC (09CA115-2), T4-3102 exhibited high levels of resistance (ITs = 0; to 1–), whereas Rusty was fully susceptible (ITs = 3+ to 4; Figure 1B). We evaluated another subset of 145 F_2_ individuals from the same population with race BCCBC and found some F_2_ plants with intermediate reactions (Supplementary Figure S1), likely due to additional minor *Sr* gene(s) in T4-3102 resistant to this race. We converted the *Pgt* reactions into two genotypic classes for the mapping purposes: ITs ranging from “0” to “2–” were considered as resistant and ITs from “3+” to “4” as susceptible [20 plants with intermediate reactions (ITs = “2+” to “3”) were discarded in the classification]. Among the 125 F_2_ plants showing clear phenotypic segregation, we observed 97 resistant plants and 28 susceptible ones, which did not deviate from the expected 3:1 segregation ratio for a single dominant gene (χ^2^ = 0.45, *P* = 0.50).

### Mapping of a Stem Rust Resistance Gene on Chromosome Arm 2BL

**Figure 2:**
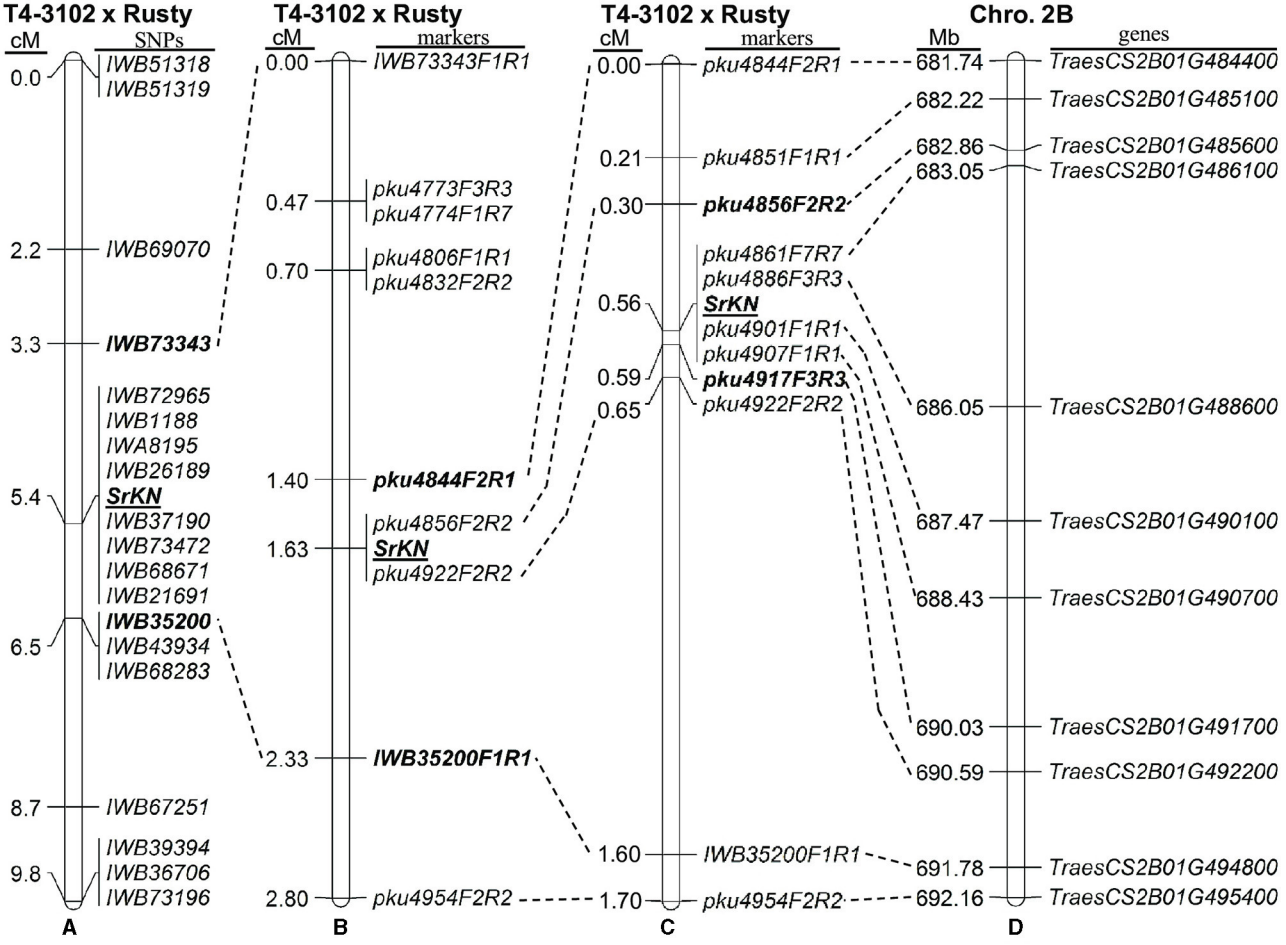
Genetic maps of *SrKN* on chromosome arm 2BL. **(A)** Initial map based on 46 F_2_ plants and wheat 90K single nucleotide polymorphism (SNP) iSelect array; **(B)** Genetic map based on 215 F_2_ plants and 10 molecular markers; **(C)** High-density map based on 1,683 F_2_ plants and 11 molecular markers; **(D)** Colinear region in the sequenced Chinese Spring (CS) reference genome (RefSeqv1.0).

For the initial mapping, we genotyped the parental lines and the more susceptible and resistant lines from the two sub-populations evaluated with TRTTF and BCCBC using the 90K SNP iSelect Illumina assay. For the 90 F_2_ plants inoculated with race TRTTF, we genotyped 10 resistant and 10 susceptible plants, whereas, for the 125 F_2_ plants challenged with race BCCBC, we genotyped 13 resistant and 13 susceptible phenotypes. We identified 4,652 polymorphic SNPs with <20% missing data between T4-3102 and Rusty. Of those, we detected 19 SNPs (Table 1) on the long arm of chromosome 2B that were significantly correlated with both TRTTF and BCCBC resistance phenotypes, suggesting that the same *Sr* gene was conferring resistance to both races. These SNPs were distributed from 106.5 to 119.6 cM (Table 1) in the 90K consensus map of chromosome 2B (https://triticeaetoolbox.org/wheat/). Based on a preliminary linkage map constructed using the 46 genotyped plants (Figure 2A), the TRTTF- and BCCBC-resistance gene *SrKN* was mapped to a 3.2 cM region between the SNPs *IWB73343* and *IWB35200*.

**Table 1:** The single nucleotide polymorphisms (SNPs) linked with *SrKN* and their locations in the Chinese Spring (CS) reference genome RefSeq v1.0 coordinates.

**SNP id**	**SNP Name**	**Chr**.	**Allele**	**Re-scaled distance cM^a^**	**Location in RefSeq v1.0 (bp)**
*IWB51318*	Ra_c18654_239	2B	A/G	106.563	chr2B:632381106
*IWB51319*	Ra_c18654_370	2B	G/T	106.563	chr2B:632381287
*IWB69070*	Tdurum_contig25423_72	2B	C/T	108.453	chr2B:653914722
*IWB73343*	Tdurum_contig76090_916	2B	A/G	109.526	chr2B:666482800
*IWB72965*	Tdurum_contig63945_206	2B	A/C	110.873	chr2B:682848528
*IWB1188*	BobWhite_c18540_351	2B	C/T	119.071	chr2B:682848604
*IWA8195*	IWA8195	2B	C/T	119.071	chr2B:682851442
*IWB26189*	Excalibur_c40976_111	2B	A/C	109.245	chr2B:683027002
*IWB37190*	JD_c2156_2040	2B	A/G	110.873	chr2B:683029851
*IWB73472*	Tdurum_contig80351_311	2B	G/T	110.873	chr2B:683047326
*IWB68671*	Tdurum_contig17626_268	2B	A/G	109.245	chr2B:683175627
*IWB21691*	Excalibur_c10634_156	2B	A/G	112.451	chr2B:689485124
*IWB35200*	IAAV6424	2B	T/C	112.868	chr2B:691780716
*IWB43934*	Kukri_c31059_130	2B	T/C	112.946	chr2B:692468899
*IWB68283*	Tdurum_contig14707_251	2B	T/C	115.008	chr2B:692712251
*IWB67251*	Tdurum_contig11711_384	2B	A/G	115.862	chr2B:714785476
*IWB39394*	Ku_c4168_1399	2B	T/C	116.819	chr2B:727205329
*IWB36706*	Jagger_c6844_121	2B	T/C	119.071	chr2B:730191209
*IWB73196*	Tdurum_contig71365_233	2B	A/G	119.613	chr2B:738410414

a *Re-scaled distances for the markers are from https://triticeaetoolbox.org/wheat/*.

*The details of the SNP markers are available online (https://triticeaetoolbox.org/wheat/)*.

Using the sequences flanking the target SNPs, we performed BLASTN searches against the reference genome of hexaploid wheat CS (RefSeqv1.0). This defined a candidate gene region on the long arm of chromosome 2B extending from 666.5 to 691.8 Mb (Table 1). Since flanking SNP markers *IWB73343* and *IWB35200* were located within the wheat genes *TraesCS2B01G470100* and *TraesCS2B01G494800*, we developed B-genome specific PCR markers *IWB73343F1R1* and *IWB35200F1R1* (Table 2) using these two genes sequences. Using these markers, we genotyped the 215 F_2_ plants previously phenotyped with races TRTTF (90 plants) and BCCBC (125 plants), which provided a better estimate of the genetic length of the candidate region (2.3 cM). Based on this new data, *SrKN* was mapped 1.6 cM distal to *IWB73343F1R1* and 0.7 cM proximal to *IWB35200F1R1* (Figure 2B). We then developed molecular markers for seven additional genes within the candidate gene region (Figure 2B; Table 2) and mapped *SrKN* between CAPS markers *pku4844F2R1* and *IWB35200F1R1*, and completely linked to markers *pku4856F2R2* and *pku4922F2R2* (Figure 2B).

**Table 2:** The primers used in the present study.

**Markers**	**Marker type**	**Forward primer (5^**′**^-3^**′**^)**	**Reverse primer (5^**′**^-3^**′**^)**	**Restriction enzyme**	**Ann.T (^**°**^C)**	**Expected size (bp)^#^**
*IWB73343F1R1*	dominant	AGAATACAGAAATAAGGAGGTGC	GATGTTTAAGAGCTGGTAAACACT	–	51	374
*pku4773F3R3*	CAPS	CGGGGATTAGACTTATTTCCTG	GGTTAGCTCTGCATCATAACTTCA	*Ava*II	55	890
*pku4774F1R7*	CAPS	GAGATCATCCAGTTAGTAACGT	TATATTCTGCTTGCTGGGT	*SSp*I-HF	50	1,319
*pku4806F1R1*	dominant	AGAAATAGCCCAGGGAATAGG	ATCCTGAATCTGTGGCCGTCT	–	58	319
*pku4832F2R2*	CAPS	CTGGCCTTGGAAGTTTACC	CCTACAGCTAACTAGATGAACCTTA	*SfaN*I	52	673
*pku4844F2R1*	CAPS	TTGATCTCGGTGAAGAAGC	CCCACCAAATTAAGTCGTT	*Stu*I	50	958
*pku4851F1R1*	Sequencing	GATTACTACTCCAATACTTCCG	AAGTCCTTTCCCTTGCTGT	–	59	520
*pku4856F2R2*	CAPS	TCCTTGGTCATCGAGATAGG	GCTGGTCAAAGCTTGAATTTG	*Mse*I	52	390
*pku4861F7R7*	CAPS	CTTTGGGGGTAATAGACACTCTA	TGATTCCCACCCTGTTCTTG	*BsmA*I	54	429
*pku4886F3R3*	InDel	CCAACTGTGCTGGTTCCTT	TTGCTTTGATTGGCTGTCTAA	–	52	640/712
*pku4901F1R1*	Sequencing	GTCTTTCAGTTATGCACTTTATTAT	TGTAGGAGCCAAGCGTATT	–	52	1,300
*pku4907F1R1*	CAPS	TTCCAGCTTTATGTACGTGTAGT	TCCATTCAGGACGAAGTGC	*Hha*I	58	671
*pku4917F3R3*	CAPS	TCAATAGGCTGAGATAACTGC	TGTGTACCCAAAGAAGAAGG	*Hha*I	52	1,400
*pku4922F2R2*	CAPS	AACCTGGTCCGTGAAAGA	AGTTGCGAAATCCCTTGCC	*Ase*I	53	1,039
*IWB35200F1R1*	CAPS	TTAGAACAAAGAGAAAATCCAGC	TCAAGCCCCTGACTAGCAGT	*HpyCH4*III	56	757
*pku4954F2R2*	CAPS	CCAGGTTCACCCTCAACTTC	CAGCTTTCTTTCACACAGCAA	*BsmA*I	57	587
*wmc332*	SSR	CATTTACAAAGCGCATGAAGCC	GAAAACTTTGGGAACAAGAGCA	–	61	169

*CAPS, cleaved amplified polymorphic sequence; SSR, simple sequence repeat; InDel, insertion/deletion*.

# *The expected size corresponds to the original size without digestion. For the InDel marker, the former represents the size in Kronos and the latter represents in Rusty*.

To define the position of *SrKN* more precisely, we screened another 1,468 plants from four selected segregating F_3_ families with the new flanking markers *pku4844F2R1* and *IWB35200F1R1*. The distance between these two flanking markers was estimated to be 1.6 cM based on the 50 plants with recombination events identified in this screen and the four recombinants identified between these same markers in the previous 215 plants. For these 54 informative F_3_ families, we performed progeny tests (25 plants per family) with races BCCBC and 34MKGQM in growth chambers. Using these new recombination events and six new markers developed in this region (Table 2; Supplementary Figure S2), we further delimited the *SrKN* candidate region to a 0.29-cM interval (7.2-Mb, CS RefSeq v1.0 coordinates) flanked on the proximal side by marker *pku4856F2R2* (0.26 cM) and on the distal side by *pku4917F3R3* (0.03 cM) (Figure 2C).

### Candidate Genes for *SrKN* Within the Colinear Regions of Tetraploid and Hexaploid Wheat Genomes

The 0.29 cM candidate region between the markers *pku4856F2R2* and *pku4917F3R3* defines a 5.6-Mb region in *T. turgidum* ssp. *durum* cv. Svevo (672.6–678.2 Mb, Supplementary Table S2) and a 7.2-Mb region in *T. aestivum* cv. CS (682.8–690.0 Mb, Figure 2D; Supplementary Table S3). These candidate gene regions include 52 annotated high-confidence genes in Svevo (*TRITD2Bv1G223060*–*TRITD2Bv1G224370*) and 59 in Chinese Spring (*TraesCS2B02G485600*–*TraesCS2B02G491700*) (Figure 2D). These genes included nine typical NBS-LRR (NLR) in Svevo and six in CS, which is of particular interest for this project because NLRs are the most frequent gene class associated with disease resistance in the plants.

Among the 52 genes annotated in the candidate gene region in the Svevo genome, we found that 35 of them were expressed in Kronos, based on BLASTN searches in the published Kronos transcriptome database (Krasileva et al., 2013) (https://dubcovskylab.ucdavis.edu/wheat_blast). The expressed genes include seven of the nine annotated NLR genes (*TRITD2Bv1G223210, TRITD2Bv1G223370, TRITD2Bv1G223450, TRITD2Bv1G223460, TRITD2Bv1G223490, TRITD2Bv1G223550*, and *TRITD2Bv1G223640*). We designed two to four pairs of primers for each of the seven expressed NLR genes and all of them amplified the expected bands in Kronos genomic DNA (Supplementary Table S4). By contrast, only two of the 22 primers pairs (*TRI2B223210F7R7* and *TRI2B223490F3R3*) amplified products in Rusty, suggesting that these NLRs may be absent in Rusty (or partially deleted). To rule out the possibility that the lack of amplification in Rusty was caused by degraded DNA, the same genomic DNAs were tested with the primers *pku4856F2R2, pku4861F7R7, pku4886F3R3, pku4907F1R1*, and *pku4917F3R3* (Table 2) and the expected bands were obtained in Rusty (Supplementary Table S4). We cannot rule out the possibility that some of the primers that failed to amplify the Rusty genomic DNA were caused by polymorphisms in the primer regions rather than by the absence of the genes.

### Comparison of Mapping Positions and Resistance Profiles of *SrKN, Sr9*, and *Sr28* Resistance Genes Located on Chromosome Arm 2BL

#### Comparison of Map Locations

**Figure 3:**
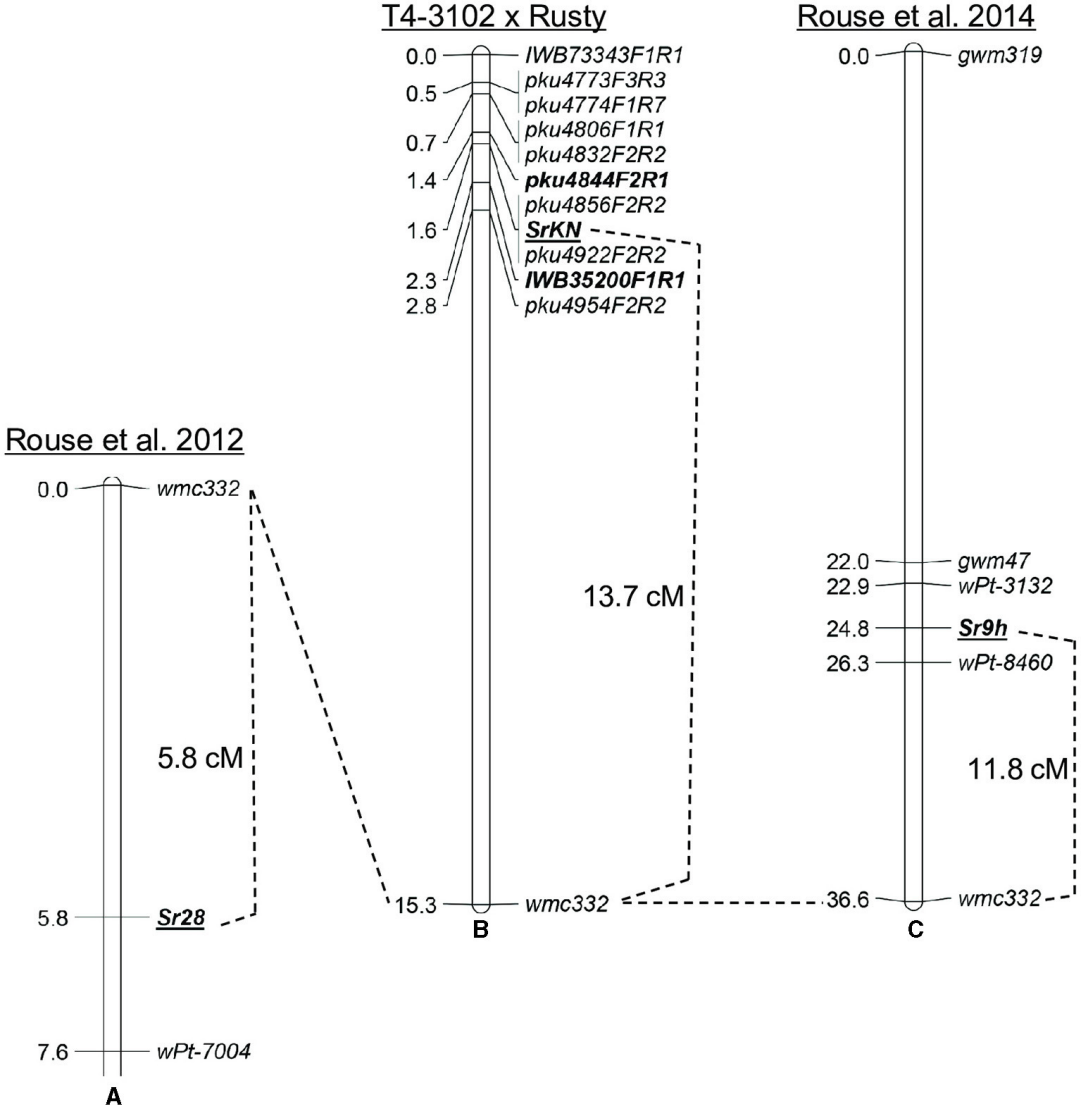
Relative map position of *Sr28, SrKN*, and *Sr9h*. **(A)** Genetic map of *Sr28* derived from the population LMPG-6 × SD 1691 (Rouse et al., 2012); **(B)** Genetic map of *SrKN* from the population T4-3102 × Rusty in the present study; **(C)** Genetic map of *Sr9h* from the population Gabo 56 × CS (Rouse et al., 2014).

Two wheat stem rust resistance genes, *Sr9* and *Sr28*, were previously mapped close to *SrKN* on chromosome arm 2BL (Rouse et al., 2012, 2014; Yu et al., 2014). To compare their relative map positions, we used the simple sequence repeat (SSR) marker *wmc332* that was previously shown to be linked to *Sr9* and *Sr28* (Rouse et al., 2012, 2014). We mapped *wmc332* in the population of 215 F_2_ plants mentioned above and found that *SrKN* is located 13.7 cM proximal to this marker (Figure 3A), whereas *Sr28* was mapped roughly 5.8 cM distal to the same marker (Figure 3B) (Rouse et al., 2012). These results suggest that *SrKN* and *Sr28* are two different loci located about 20 cM apart (Figure 3).

The *Sr9* gene has multiple alleles that include *Sr9a, Sr9b, Sr9d, Sr9e, Sr9f*, *Sr9g*, and *Sr9h* (McIntosh et al., 2013; Rouse et al., 2014). The gene *Sr9h* was mapped 11.8 cM proximal to *wmc332* (Rouse et al., 2014), indicating that *SrKN* and *Sr9h* loci can be close to each other or represent the same gene (Figures 3B,C). To test this hypothesis, we performed an allelism test using a BC_2_F_2_ monogenic line for *Sr9e* derived from the durum wheat variety Vernal (Vernal-BF9e) crossed by the monogenic *SrKN* line Td31-5R (as shown in Material and methods for the development of these lines). None of the 470 F_2_ plants generated from this cross inoculated with *Pgt* race 34MKGQM showed a susceptible reaction suggesting that *Sr9* and *SrKN* are allelic.

#### Comparison of Resistance Profiles

Inoculation of the *SrKN* monogenic line Td31-5R and its sister line Td31-7S lacking *SrKN* with different *Pgt* races showed that this gene is ineffective against the evaluated races TTKSK, TKTTF, and JRCQC but confers resistance to the races BCCBC, TRTTF, 21C3CTTTM, 34MKGQM, 34MTGSM, and 34C3RTGQM (Supplementary Figure S3a; Table 3). *Sr28* was evaluated against race TRTTF and another four races from China and was not effective against any of them (Li et al., 2016, 2018; Babiker et al., 2017) (Table 3), supporting the conclusion from the genetic data that *Sr28* and *SrKN* are two different genes.

**Table 3:** The resistance profiles of *Sr9* alleles, *Sr28*, and *SrKN* to multiple *Puccinia graminis* f. sp. *tritici* races.

**Genes**	***Puccinia graminis*** **f. sp**. ***tritici*** **races (isolates)**								
	**TRTTF (06YEM34-1)**	**BCCBC (09CA115-2)**	**TTKSK (04KEN156/04)**	**TKTTF (13ETH18-1)**	**JRCQC (09ETH08-3)**	**21C3CTTTM (20GH13)**	**34MKGQM (20IAL06)**	**34MTGSM (20GSA1)**	**34C3RTGQM (20IAL32)**
*Sr9a*	S	R	S	S	S	S	S	S	S
*Sr9b*	S	R	S	S	R	S	S	S	S
*Sr9d*	S	R	S	S	S	S	S	S	S
*Sr9e*	R^a^	R	S	S	S	R	R	R	R
*Sr9f*	S	NA	S	S	S	S	S	S	NA
*Sr9g*	S	S	S	S	S	S	S	S	S
*Sr9h*	S	NA	R	S	S	NA	NA	NA	NA
*Sr28*	S	R	R	S	NA	S	S	S	S
*SrKN*	R	R	S	S	S	R	R	R	R

*R, resistant; S, susceptible; NA, not available*.

a *Sr9e was initially reported to be susceptible to TRTTF (Olivera et al., 2012) but a more recent report suggested that it confers partial resistance to this race (Saini et al., 2018)*.

Among the different *Sr9* alleles, the most similar profile to *SrKN* was found for *Sr9e*. These two genes showed similar reactions for eight of the nine races tested, and differed only for race TRTTF for which *SrKN* was resistant and *Sr9e* was reported to be susceptible (Olivera et al., 2012) (Table 3). However, a more recent report suggested that *Sr9e* confers partial resistance to race TRTTF (Saini et al., 2018), which would result in identical profiles between *SrKN* and *Sr9e*. We also challenged the hexaploid line Vernstein, which is known to carry the *Sr9e* allele, with the Chinese race 34MKGQM and found a similar level of *Pgt* resistance to that conferred by lines Vernal-BF9e and Td31-5R (Supplementary Figure S3).

The alleles *Sr9a, Sr9b, Sr9d*, and *Sr9g* differ from *SrKN* by their susceptibility to races TRTTF, 34C3RTGQM, 34MKGQM, 34MTGSM, and 21C3CTTTM (Table 3). In addition, the *Sr9f* allele was shown to be ineffective to 21C3CTTTM, 34MKGQM, and 34MTGSM (Li et al., 2018) suggesting that *Sr9* alleles *Sr9a, Sr9b, Sr9d, Sr9f*, and *Sr9g* have a very different resistance profile than *SrKN*. Finally, *Sr9h* was shown to be resistant to TTKSK (Rouse et al., 2014), whereas *SrKN* was ineffective against this race. In summary, based on the currently available information, the most similar *Sr9* allele to *SrKN* is *Sr9e*.

### Detection of *SrKN* and/or *Sr9e* Resistance Based on the Haplotype of Linked Markers

To determinate the value of the haplotype defined by the two flanking markers and three completely linked polymorphisms, we developed one Insertion/deletion (InDel) and four CAPS markers and used them to screen a panel of durum and bread wheat lines. The same lines were evaluated with *Pgt* race 34MKGQM (Supplementary Table S5). T4-3102 (*SrKN*) and Vernal (*Sr9e*) showed an identical haplotype indicating that these five markers are not sufficient to differentiate these genes/alleles. By contrast, Rusty differed from T4-3102 in all the five polymorphisms (Supplementary Table S5) indicating a very different haplotype.

Among the 20 durum lines compared with T4-3102 (*SrKN*), Vernal (*Sr9e*), and Rusty, only Svevo and Langdon showed a haplotype identical to *SrKN* and *Sr9e*. These four lines also displayed a similar resistance response against race 34MKGQM, suggesting that Svevo and Langdon might carry *SrKN* or *Sr9e*. Eleven lines carried the Rusty haplotype and were susceptible to 34MKGQM (Supplementary Table S5), confirming the absence of *SrKN* in these lines. Among the other seven lines, four showed the same haplotype as Rusty but higher levels of resistance than T4-3102 suggesting the presence of other *Sr* genes. The last three lines showed different haplotypes from the three control lines and resistance levels higher than *SrKN* or *Sr9e*, also suggesting the presence of other *Sr* genes (Supplementary Table S5). Indeed, four of them were confirmed to carry the cloned gene *Sr13* and the line PI 94701 was known to possess the resistance gene *Srdp2* (Rondon et al., 1966) (Supplementary Table S5).

Among the 16 hexaploid wheat lines analyzed, we detected the *SrKN*/*Sr9e* haplotype in Vernstein (*Sr9e*), Cn*Sr9g*, and I*Sr9a*-Ra, suggesting that these markers were not able to differentiate *SrKN* from *Sr9g* and *Sr9a*. All the tested hexaploid wheat lines were susceptible to race 34MKGQM except Vernstein (Supplementary Table S5). In summary, the five polymorphisms seem to be useful to predict the presence of the *SrKN* allele, but they cannot differentiate it from the more susceptible alleles *Sr9g* and *Sr9a*.

### Transfer of Stem Rust Resistance to Hexaploid Wheat Background

**Figure 4:**
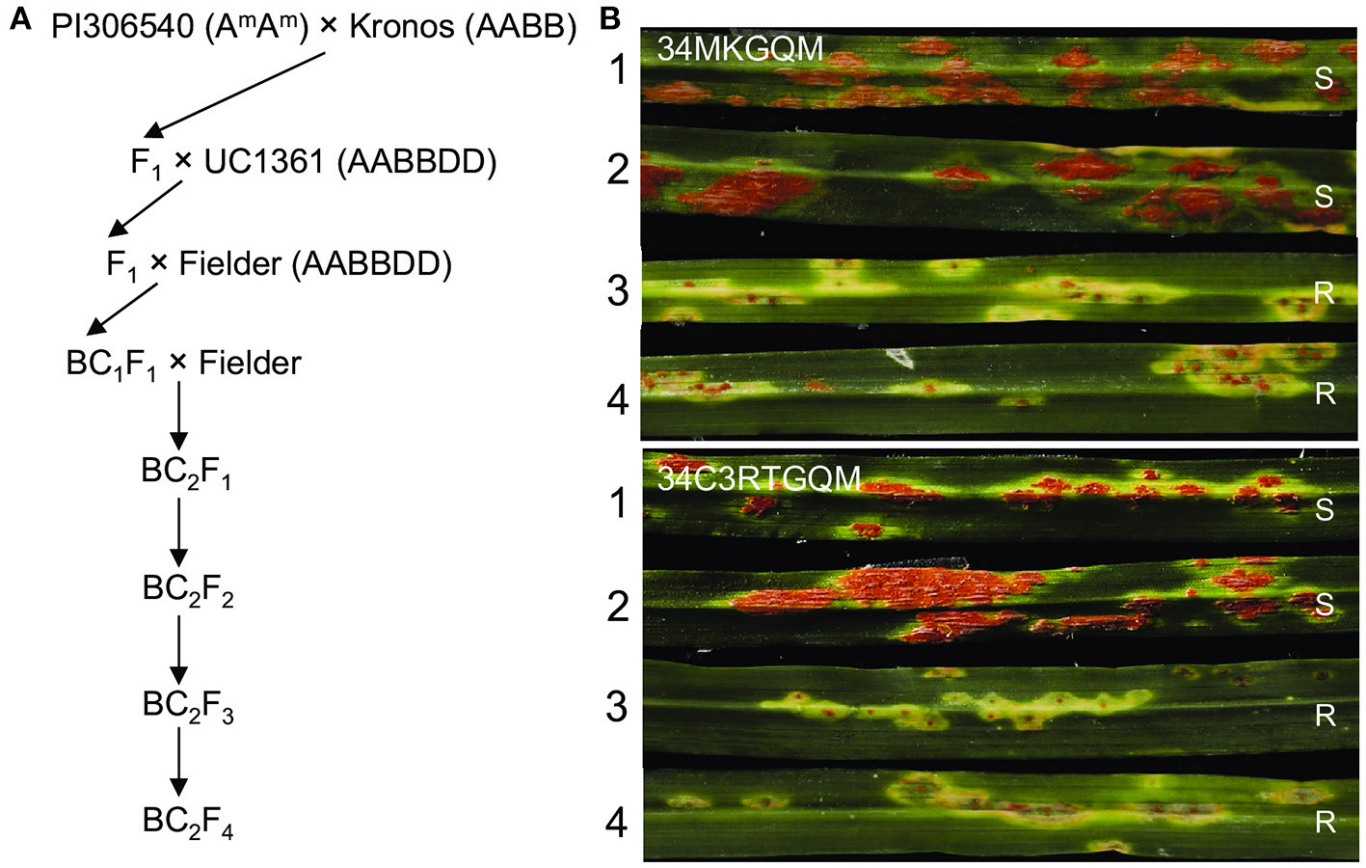
Introgression of *SrKN* into a hexaploid wheat background. **(A)** Procedure involved in the generation of the *SrKN* introgression into common wheat. Flanking markers *pku4856F2R2* and *pku4917F3R3* and completely linked markers *pku4861F7R7* and *pku4886F3R3* were used to confirm the presence of Kronos chromatin during crosses. **(B)** Infection types (ITs) from the BC_2_F_3_ plants were homozygous for the resistant *SrKN* allele (+*SrKN*) and the plants lacking *SrKN* (–*SrKN*). Two *Pgt* races 34MKGQM and 34C3RTGQM were used to evaluate. 1–2, BC_2_F_3_ plants lacking the resistant *SrKN* allele; 3–4, BC_2_F_3_ plants homozygous for the resistant *SrKN* allele. R, resistant; S, susceptible.

To transfer the resistance gene *SrKN* to hexaploid wheat, we took advantage of the crosses previously used to transfer several *T. monococcum* resistance genes into hexaploid wheat (Figure 4). We first crossed Kronos with the *T. monococcum* accession PI 306540 (A^m^A^m^), which carries the additional stem rust resistance genes *Sr21, Sr60, SrTm4*, and *SrTm5* (Briggs et al., 2015; Chen et al., 2018a,b; Chen et al., 2020). The resulting F_1_ triploid plants were crossed with common wheat variety Clear White (PVP 2004-00244) and then backcrossed two times to the hexaploid wheat line Fielder, which is susceptible to the *Pgt* races 34MKGQM and 34C3RTGQM. Four PCR markers *pku4856F2R2, pku4861F7R7, pku4886F3R3*, and *pku4917F3R3* were used to confirm the presence of the Kronos segment in the final BC_2_F_2_ lines. Markers for the other *T. monococcum* genes identified one BC_2_F_2_ plant heterozygous for *SrKN* but lacking all the other parental *Pgt* resistance genes *Sr21, Sr60, SrTm4, SrTm5* (from *T. monococcum*), and *Sr13* (from Kronos).

In the BC_2_F_3_ progeny derived from the selected BC_2_F_2_ plant, we identified eight plants homozygous for *SrKN* alone and six plants without any *Sr* genes (Supplementary Figure S4). Half of the plants from each genotype were inoculated with race 34MKGQM and the other half with 34C3RTGQM. The plants carrying *SrKN* exhibited good levels of resistance (IT = 1+) to both races, whereas plants lacking *SrKN* showed susceptible reactions (IT = 3+ to 4) to the same races (Figure 4). We are currently increasing the seeds from the plants carrying only *SrKN* to deposit them in the National Small Grain Collection in the United States and the Germplasm Bank of China.

## Discussion

### High-Density Mapping of *SrKN* and Delimitation of Its Candidate Gene Region

A previous study postulated that, in addition to *Sr13*, the durum wheat variety Kronos carries an undetermined stem rust resistance gene effective against *Pgt* race TRTTF (Zhang et al., 2017). In this study, we mapped this TRTTF-resistance gene *SrKN* within a 0.29 cM region on the distal region of chromosome arm 2BL using a high-density genetic map.

Using the published sequenced genomes of tetraploid and hexaploid wheat (The International Wheat Genome Sequencing Consortium, 2018; Maccaferri et al., 2019), we delimited the *SrKN* candidate gene region to a 5.6-Mb region in tetraploid wheat Svevo and a 7.2-Mb region in hexaploid wheat CS (Supplementary Tables S2, S3) including a cluster of NLR genes. Since NLR genes are the most frequent class associated with disease resistance in wheat and other plant species (Gassmann et al., 1999; Yuan et al., 2011; Saintenac et al., 2013; Zhang et al., 2014, 2017; Chen et al., 2018b; Li et al., 2019), we hypothesize that one of these genes could be a good candidate for *SrKN*. This hypothesis is supported by the complete linkage of this cluster to *SrKN* and by their likely absence in the susceptible parent Rusty (Supplementary Table S4). Similar to the *SrKN* candidate region, deletions, rearrangements, and duplications of NLR genes have been described for other cloned wheat NLR genes involved in resistance to *Pgt*, such as *Sr21* and *Sr13* (Zhang et al., 2017; Chen et al., 2018b). To determine if these NLR genes were required for resistance to TRTTF, we are currently testing truncation mutations for each gene from the published database of sequenced ethyl methane sulfonate (EMS) mutations in Kronos (Krasileva et al., 2017).

Since we do not have a contiguous sequence of the Kronos genome, we cannot rule out the possibility of additional NLR genes present in Kronos that are absent in the Svevo reference genome. However, this is unlikely because the sequences of all the genes in the candidate region (Supplementary Table S2, from start to stop codons) were 100% identical between Kronos and Svevo, suggesting that these two varieties have a very similar or identical haplotype in this region. In addition, Svevo has a similar resistance response against race 34MKGQM (Supplementary Figure S3; Supplementary Table S5). Taken together, these results suggest that Svevo may also carry *SrKN* or *Sr9e*. If this is confirmed, the availability of the Svevo genome can accelerate the identification of the causal gene.

### Relationship Between *SrKN* and Other *Sr* Genes on Chromosome Arm 2BL

In addition to gene *SrKN*, previous studies have identified other four stem rust resistance loci on chromosome arm 2BL (*Sr9, Sr16, Sr28*, and *Sr47*) (McIntosh et al., 1995; Klindworth et al., 2012; Rouse et al., 2012). Among these genes, *Sr47* confers resistance to race TTKSK and was transferred from *Aegilops speltoides* Tausch into polyploid wheat (Klindworth et al., 2012). Gene *Sr16* is not effective against race TRTTF (Singh et al., 2015), and *Sr28* showed a very different resistance profile to *SrKN* in this study (Table 3). The genetic analysis using a shared SSR marker indicates that *Sr28* is located about 20 cM distal to *SrKN* (Figure 3). Gene *Sr16* was placed approximately 34 cM distal to *Sr28* by using monosomic analysis (~54 cM distal to *SrKN*) (McIntosh, 1978; Hiebert et al., 2010). Based on these data, we concluded that *SrKN* is different from genes *Sr16, Sr28*, and *Sr47*.

Conflictive or inconclusive results were reported regarding the mapping locations of *Sr9*. Gene *Sr9e* was initially mapped approximately 0.7 cM proximal to SSR marker *gwm47* (685,759,255 bp, RefSeq v1.0 coordinates) (Bhavani et al., 2008). By contrast, another *Sr9* allele, *Sr9h*, was mapped 2.8 cM distal to the same marker (Rouse et al., 2014). A recent study showed that the *Sr9* locus is located within a region of chromosome 2B between 665.7 and 720.5 Mb in the reference genome of CS (RefSeq v1.0) (Aoun et al., 2019), which includes our proposed candidate region for *SrKN* (682.9–690.0 Mb). In addition, another recent study has mapped a TRTTF resistance quantitative trait locus (QTL) derived from tetraploid wheat accession Langdon on chromosome 2BL, which was designated as *QSr.rwg-2B.2* and was hypothesized to be *Sr9e* (Sharma et al., 2021). Although the authors suggested that this QTL was mapped between the SNP markers *IWB71742* (738.3 Mb, RefSeq v1.0) and *IWB73196* (738.4 Mb), this QTL extends to a much larger region from *IWB3657* (593.6 Mb) to *IWB11280* (750.0 Mb) that includes our candidate gene region. Previous studies postulated that Langdon carries *Sr9e* (Luig, 1983; Singh et al., 1992), a conclusion supported by our analysis of the Langdon haplotype in the *Sr9e* region, which is identical to the one we found in Kronos (Supplementary Table S5).

We initially thought *SrKN* and *Sr9e* were different genes because *Sr9e* was reported to be susceptible to race TRTTF (Olivera et al., 2012) and *SrKN* is not. However, more recent reports suggested that *Sr9e* conferred partial resistance to race TRTTF (Saini et al., 2018; Sharma et al., 2021). If this last result is confirmed to be correct, then the resistance profiles of *SrKN* and *Sr9e* would be identical. Taken together, the allelism test and the similar resistance profiles (Table 3) suggest (but do not demonstrate) that *SrKN* and *Sr9e* might be the same gene.

### Introgression of *SrKN* Into Hexaploid Wheat and Its Utilization in Breeding

As durum and bread wheat have common A and B genomes, it is relatively easy to introgress important genes into bread wheat from *T. durum*. Several rust resistance genes have been identified and transferred from durum to hexaploid wheat, including the stripe rust resistance genes *Yr5* (Zhang et al., 2009), *Yr53* (Xu et al., 2013), *Yr64*, and *Yr65* (Cheng et al., 2014); the leaf rust resistance genes *Lr23* (McIntosh et al., 1995; Sibikeev et al., 2020), *Lr61* (Herrera-Foessel et al., 2008), and *Lr79* (Qureshi et al., 2018); and the stem rust resistance genes *Sr12* (Sheen and Snyder, 1964), *Sr13* (Simons et al., 2011; Zhang et al., 2017), and *Sr8155B1* (Nirmala et al., 2017). Using the cross of Kronos (AABB) × PI 306540 (A^m^A^m^), we successfully introgressed *SrKN* into hexaploid wheat line Fielder. The same cross was also used to introgress the stem rust resistance gene *Sr60* and *SrTm5* from diploid wheat accession PI 306540 into the common wheat lines UC12014-36 and Fielder, respectively (Chen et al., 2018a, 2020).

Although the crosses between tetraploid and hexaploid wheat can generate viable pentaploid plants, some of these crosses result in hybrid necrosis limiting their use in commercial breeding programs. Therefore, the introgression of *SrKN* into a common wheat background will facilitate the utilization of this resistance gene in common wheat breeding programs. Since *SrKN* is not effective against several virulent *Pgt* races (Zhang et al., 2017), including the Ug99 race group and race TKTTF, it is important to deploy it in combination with other *Sr* genes. Some potentially useful combinations to expand the resistance spectrum include *Sr21* (Chen et al., 2015), *SrTm5* (Chen et al., 2018a), *Sr36* (Singh et al., 2015), *Sr1RS*^*Amigo*^ (Olivera et al., 2012), and *SrTmp* (Singh et al., 2015), which are susceptible to *Pgt* race TRTTF but confer resistance to TTKSK (Ug99).

In conclusion, the high-density map of *SrKN*, the closely linked molecular markers, and the introgression of the *T. durum* segment containing this gene into hexaploid wheat will accelerate its deployment and pyramiding with other *Sr* genes.

## Data Availability Statement

Raw sequencing data of durum wheat Rusty has been deposited in NCBI's Sequence Read Archive (https://www.ncbi.nlm.nih.gov/sra, Bioproject PRJNA751176).

## Author Contributions

HongnaL and LH performed most of the experimental work. MR performed the phenotyping experiments with race TRTTF. TL contributed part of the phenotyping experiments with Chinese *Pgt* races. SP, SB, TS, JL, HongyuL, and XW contributed the mapping and primers development. WZ created the mapping population and phenotyping with race BCCBC. SC analyzed the data and wrote the first version of the manuscript. SC and JD proposed and supervised the project, obtained the funding, and generated the final version of the paper. All authors revised the manuscript and provided suggestions.

## Funding

Work at JD laboratory was supported by the Howard Hughes Medical Institute and by the Agriculture and Food Research Initiative Competitive Grant 2017-67007-25939 (WheatCAP) from the USDA National Institute of Food and Agriculture (NIFA). Work at SC laboratory was supported by the Provincial Technology Innovation Program of Shandong and by the State Key Laboratory of North China Crop Improvement and Regulation (NCCIR2020KF-4). Work at the USDA-ARS was supported by the USDA-ARS National Plant Disease Recovery System. Work at the XW laboratory was supported by the Provincial Natural Science Foundation of Hebei (C2021204008).

## Conflict of Interest

The authors declare that the research was conducted in the absence of any commercial or financial relationships that could be construed as a potential conflict of interest.

## Publisher's Note

All claims expressed in this article are solely those of the authors and do not necessarily represent those of their affiliated organizations, or those of the publisher, the editors and the reviewers. Any product that may be evaluated in this article, or claim that may be made by its manufacturer, is not guaranteed or endorsed by the publisher.
