# Supplementary material for: Mapping and Characterization of a Wheat Stem Rust Resistance Gene in Durum Wheat “Kronos”
Source: Front Plant Sci. 2021 Oct 15;12:751398. doi: 10.3389/fpls.2021.751398 (PMC8555631; doi:10.3389/fpls.2021.751398)
Supplement: Supplementary file 1 [file Data_Sheet_1.PDF]

## Supplementary Figures

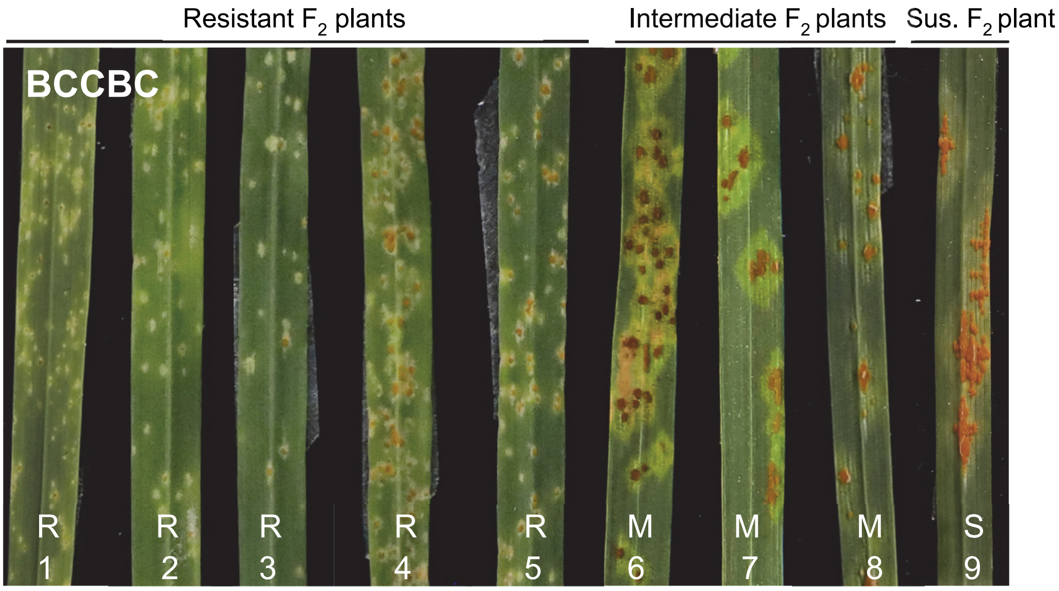


**Supplementary Fig. S1** Reactions to *Pgt* race BCCBC in a subset of 145 $F_2$ plants. Infection types ranging from '0;' to '2-' were considered as resistant (plants 1-5, Resistant $F_2$ plants) and ITs from '3+' to '4' as susceptible (plant 9, Sus. $F_2$ plant), but some $F_2$ plants showed intermediate reactions (plants 6-8, Intermediate $F_2$ plants). R, resistant; S, susceptible; M, intermediate type. Sus., susceptible.

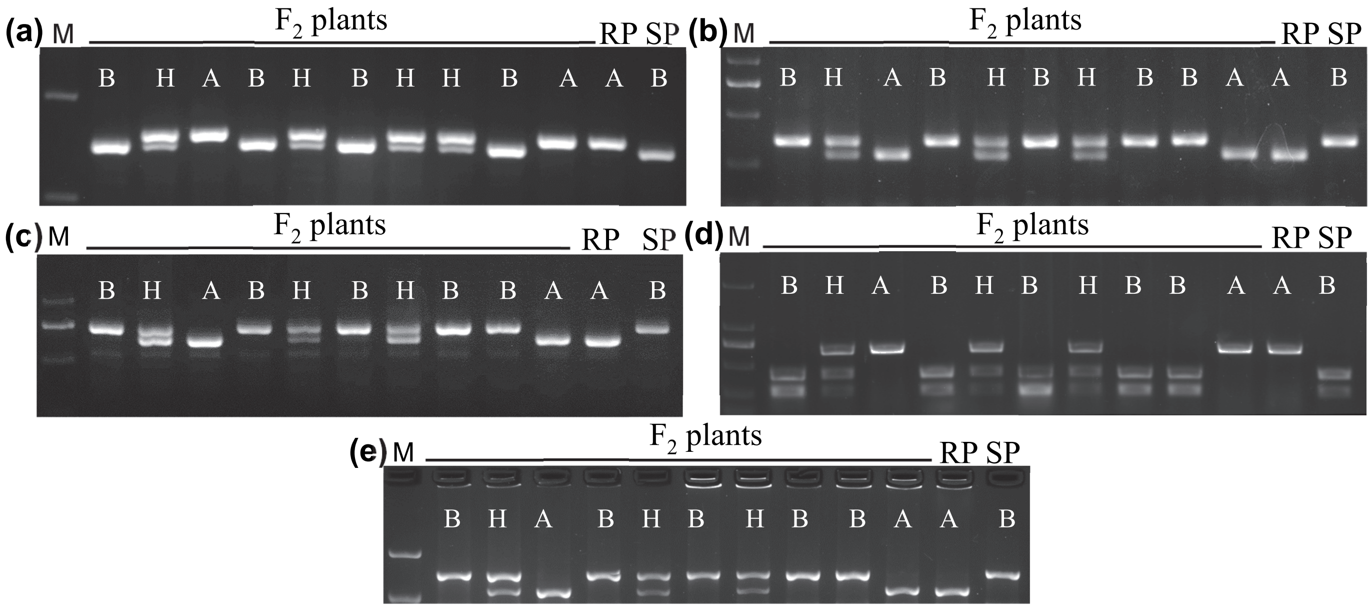


**Supplementary Fig. S2** Genotypes of $F_2$ plants derived from the cross between *Pgt* resistant line T4-3102 and susceptible accession Rusty. (a) $F_2$ plants genotyped with CAPS marker *pku4856F2R2* digested with *Mse*I; (b) $F_2$ plants genotyped with CAPS marker *pku4861F7R7* digested with *BsmA*I; (c) $F_2$ plants genotyped with InDel marker *pku4886F3R3*; (d) $F_2$ plants genotyped with CAPS marker *pku4907F1R1* digested with *Hha*I; (e) $F_2$ plants genotyped with CAPS marker *pku4917F3R3* digested with *Hha*I. Marker *pku4901F1R1* was not showed here since it is a Sanger sequencing marker. RP = resistant parent (T4-3102); SP = susceptible parent (Rusty); A, the band corresponds to the resistant allele; B, the band corresponds to the susceptible allele; H, heterozygous.

(a)

(b)

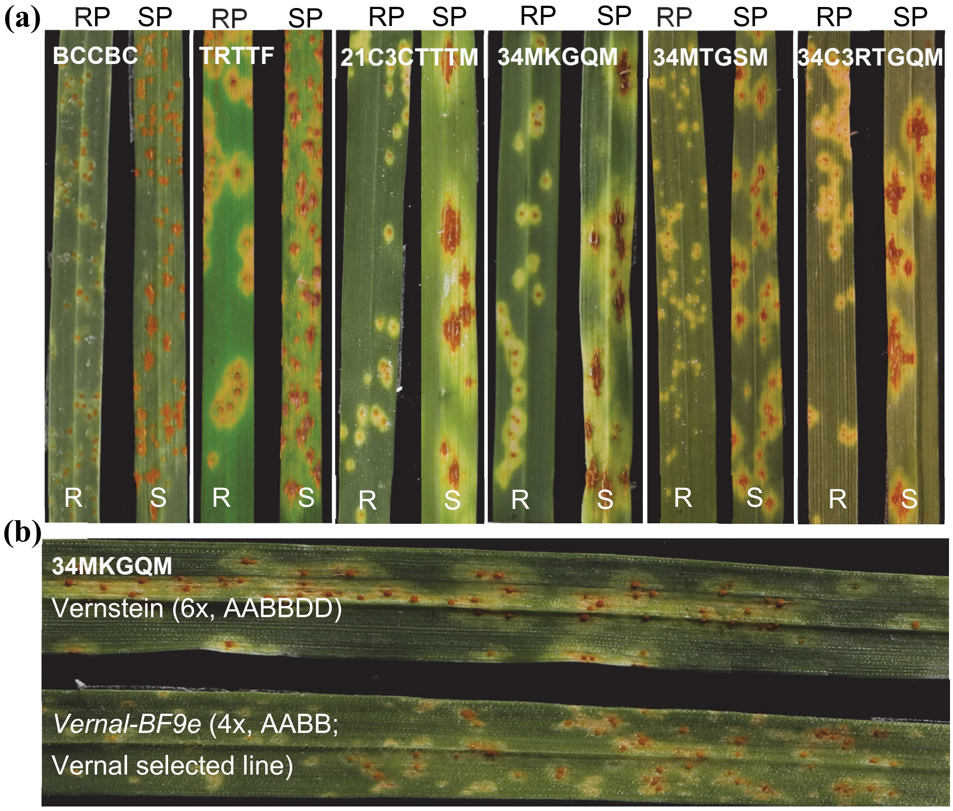


**Supplementary Fig. S3** Reactions to several *Pgt* races. (a) Infection types of two parents in response to *Pgt* races BCCBC, TRTTF, 21C3CTTTM, 34MKGQM, 34MTGSM and 34C3RTGQM. (b) Infection types of wheat lines Vernstein (*Sr9e*, 6x, AABBDD) and Vernal-BF9e (*Sr9e*, 4x, AABB) in response to race 34MKGQM. RP = resistant line, Td31-5R (*SrKN*); SP = susceptible line, Td31-7S (no *Sr* genes); R, resistant; S, susceptible.

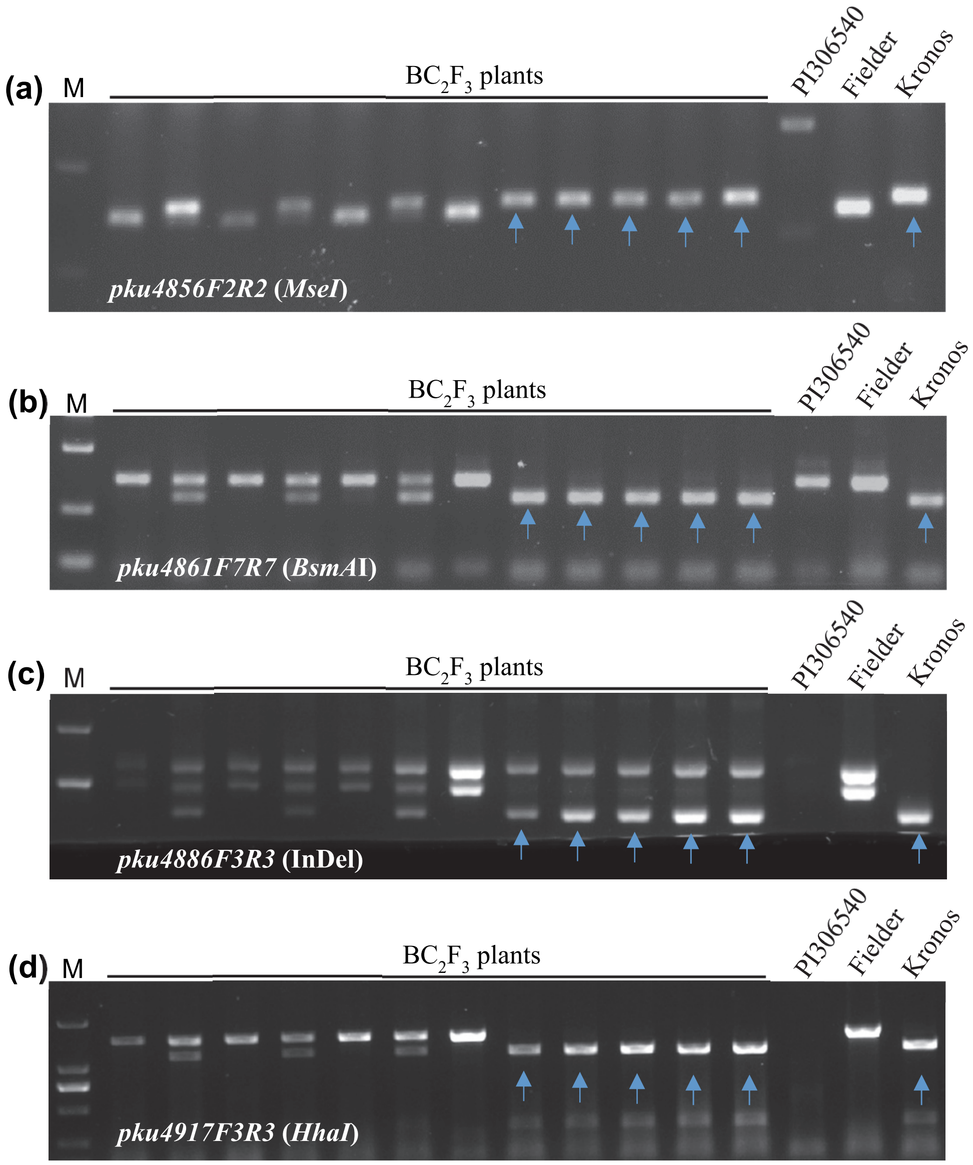


**Supplementary Fig. S4** Genotyping of selected $BC_2F_3$ introgression plants with PCR markers *pku4856F2R2*, *pku4917F3R3*, *pku4861F7R7* and *pku4886F3R3*. (a) plants genotyped with CAPS marker *pku4856F2R2* digested with *Mse*I; (b) plants genotyped with CAPS marker *pku4861F7R7* digested with *BsmA*I; (c) plants genotyped with InDel marker *pku4886F3R3*; (d) plants genotyped with CAPS marker *pku4917F3R3* digested with *Hha*I. *T. monococcum* line PI 306540, *T. durum* line Kronos, and *T. aestivum* line Fielder were used as controls.

## Supplementary Tables

**Table S1.** The avirulence / virulence formulae of the *Pgt* races used in this study.

| Race (isolate) | Origin | Avirulence | Virulence |
|---|---|---|---|
| TRTTF (06YEM34-1)[$] | Yemen | *Sr8a* ***Sr9e***[**] *24 31* | *Sr5 6 7b 9a 9b 9d 9g 10 11 17 21 30 36 38 Tmp* |
| BCCBC (09CA115-2)[&] | USA | *Sr5 6 7b 8a 9a 9d 9e 9b 10 11 21 24 30 31 36 38 Tmp* | *Sr9g 17 McN* |
| TTKSK (04KEN156/04)[$] | Kenya | *Sr21 24 36 Tmp* | *Sr5 6 7b 8a 9a 9b 9d 9e 9g 10 11 17 30 31 38 McN* |
| TKTTF (13ETH18-1)[$] | Ethiopia | *Sr11 24 31* | *Sr5 6 7b 8a 9a 9b 9d 9e 9g 10 17 21 30 36 38 McN Tmp* |
| JRCQC (09ETH08-3)[&] | Ethiopia | *Sr5 7b 8a 9b 10 24 30 31 36 38 Tmp* | *Sr6 9a 9d 9e 9g 11 17 21 McN* |
| 21C3CTTTM (20GH13)[*] | China | *Sr5 9e 14 19 21 22 23 26 27 31 33 35 37 38 39 47* | *Sr6 7b 8a 9a 9b 9d 9f 9g 10 11 12 13 15 16 17 18 20 24 25 28 29 30 32 34 36 Tmp McN* |
| 34MKGQM (20IAL06)[*#] | China | *Sr9e 10 11 13 14 17 18 19 21 22 23 25 26 30 31 33 34 35 36 37 38 47 Tmp* | *Sr5 6 7b 8a 9a 9b 9d 9f 9g 12 15 16 20 24 27 28 29 32 39 McN* |
| 34MTGSM (20GSA1)[*#] | China | *Sr9e 12 13 14 17 21 22 23 26 30 31 33 35 36 37 38 47 Tmp* | *Sr5 6 7b 8a 9a 9b 9d 9f 9g 10 11 15 16 18 19 20 24 25 27 28 29 32 34 39 McN* |
| 34C3RTGQM (20IAL32)[#] | China | *Sr9e 10 12 13 15 17 19 20 23 27 30 31 33 35 36 37 38 Tmp* | *Sr5 6 7b 8a 9a 9b 9d 9g 11 14 18 21 24 25 28 29 32 34 McN* |

[$], [&], [#] and [*], avirulence/virulence formulae shown here was based on the previous studies (Li et al. 2016 [#]; Chao et al. 2017 [&]; and Li et al. 2018 [*]; Chen et al. 2020 [$]);

[**], a recent report suggested that *Sr9e* confers partial resistance to race TRTTF (Saini et al., 2018).

**Table S2.** Predicted genes within the candidate region based on the genomic sequence of Svevo Rel. 1.0 pseudomolecules. NBS-LRR (NLR) genes are marked in blue.

| Gene ID in Svevo | Location (bp) | Gene annotation | Expressed genes in Kronos |
|---|---|---|---|
| *TRITD2Bv1G223060* | 2B:672554458-672565579 | ABC transporter C family protein | Yes |
| *TRITD2Bv1G223150* | 2B:672702835-672705697 | Remorin family protein | Yes |
| *TRITD2Bv1G223170* | 2B:672738211-672746358 | Serine/threonine-protein phosphatase 6 regulatory subunit 3 | Yes |
| *TRITD2Bv1G223180* | 2B:672747102-672747596 | RPM1-interacting protein 4 | No |
| *TRITD2Bv1G223190* | 2B:672748109-672748513 | Reverse transcriptase-like | No |
| *TRITD2Bv1G223200* | 2B:673027881-673032531 | RNA binding protein | Yes |
| *TRITD2Bv1G223210* | 2B:673034766-673039962 | **NBS-LRR disease resistance protein-like protein** | Yes |
| *TRITD2Bv1G223300* | 2B:673093651-673095655 | tRNA (Guanine-N(7)-)-methyltransferase | Yes |
| *TRITD2Bv1G223320* | 2B:673124815-673376536 | F-box family protein | Yes |
| *TRITD2Bv1G223360* | 2B:673647362-673648435 | F-box family protein | No |
| *TRITD2Bv1G223370* | 2B:673724510-673736451 | **Disease resistance protein RGA2** | Yes |
| *TRITD2Bv1G223390* | 2B:673738203-673739621 | B3 domain-containing protein | Yes |
| *TRITD2Bv1G223400* | 2B:673801060-673802315 | Transmembrane protein 45B | Yes |
| *TRITD2Bv1G223420* | 2B:673876680-673877520 | Rx N-terminal domain | Yes |
| *TRITD2Bv1G223440* | 2B:673879580-673953548 | **Disease resistance protein (TIR-NBS-LRR class) family** | No |
| *TRITD2Bv1G223450* | 2B:673948261-674761212 | **NBS-LRR disease resistance protein-like protein** | Yes |
| *TRITD2Bv1G223460* | 2B:673949431-673950986 | **NBS-LRR disease resistance protein-like protein** | Yes |
| *TRITD2Bv1G223480* | 2B:674101742-674102107 | Mediator of RNA polymerase II transcription subunit 12-like protein | No |
| *TRITD2Bv1G223490* | 2B:674104572-674108369 | **NBS-LRR disease resistance protein-like protein** | Yes |
| *TRITD2Bv1G223520* | 2B:674188488-674189525 | DNA topoisomerase G | No |
| *TRITD2Bv1G223550* | 2B:674578062-674584736 | **NBS-LRR disease resistance protein-like protein** | Yes |
| *TRITD2Bv1G223560* | 2B:674757015-674757756 | Rx N-terminal domain | Yes |
| *TRITD2Bv1G223570* | 2B:674789421-674789786 | Mediator of RNA polymerase II transcription subunit 12-like protein | No |
| *TRITD2Bv1G223580* | 2B:674790719-674793575 | **NBS-LRR disease resistance protein-like protein** | No |
| *TRITD2Bv1G223630* | 2B:674851732-674853123 | No conserved domain | Yes |
| *TRITD2Bv1G223640* | 2B:675046802-675051364 | **NBS-LRR disease resistance protein-like protein** | Yes |
| *TRITD2Bv1G223650* | 2B:675053065-675055821 | Transcription factor GTE4 | Yes |
| *TRITD2Bv1G223660* | 2B:675063535-675067703 | Nucleoredoxin | Yes |
| *TRITD2Bv1G223710* | 2B:675425782-675430477 | Protein kinase family protein | Yes |
| *TRITD2Bv1G223720* | 2B:675447647-675448657 | Peroxidase | Yes |
| *TRITD2Bv1G223740* | 2B:675468433-675468949 | Transcription factor | Yes |

| | | | |
|---|---|---|---|
| *TRITD2Bv1G223790* | 2B:675646819-675650886 | Amine oxidase family protein | Yes |
| *TRITD2Bv1G223860* | 2B:675806608-675808017 | HXXXD-type acyl-transferase family protein | Yes |
| *TRITD2Bv1G223930* | 2B:676443672-676444418 | Pectinesterase inhibitor domain containing protein | Yes |
| *TRITD2Bv1G223960* | 2B:676448498-676451256 | DNA polymerase III polC-type | Yes |
| *TRITD2Bv1G224010* | 2B:676596083-676597697 | DNA polymerase III polC-type | No |
| *TRITD2Bv1G224020* | 2B:676640498-676643662 | Peroxisomal (S)-2-hydroxy-acid oxidase | Yes |
| *TRITD2Bv1G224060* | 2B:676657551-676659924 | Signal recognition particle 14 kDa protein | Yes |
| *TRITD2Bv1G224070* | 2B:676660902-676663625 | Aminomethyltransferase | Yes |
| *TRITD2Bv1G224130* | 2B:676884751-676886664 | Pectin lyase-like superfamily protein | Yes |
| *TRITD2Bv1G224170* | 2B:677124437-677126431 | Polyphenol oxidase | Yes |
| *TRITD2Bv1G224180* | 2B:677153515-677154267 | F-box domain containing protein | No |
| *TRITD2Bv1G224190* | 2B:677158400-677161461 | Phospholipid scramblase | No |
| *TRITD2Bv1G224200* | 2B:677229526-677243908 | transmembrane protein%2C putative (DUF594) | Yes |
| *TRITD2Bv1G224230* | 2B:677421360-677422253 | Acidic endochitinase | No |
| *TRITD2Bv1G224240* | 2B:677426685-677427578 | Acidic endochitinase | No |
| *TRITD2Bv1G224260* | 2B:677532365-677532676 | Histone H4 | No |
| *TRITD2Bv1G224270* | 2B:677596946-677597257 | Histone H4 | No |
| *TRITD2Bv1G224290* | 2B:677607375-677609986 | CDP-diacylglycerol--inositol 3-phosphatidyltransferase | Yes |
| *TRITD2Bv1G224330* | 2B:677954573-677956355 | Pectin lyase-like superfamily protein | No |
| *TRITD2Bv1G224360* | 2B:678167787-678168188 | mechanosensitive channel of small conductance-like 10 G | No |
| *TRITD2Bv1G224370* | 2B:678173420-678181177 | Starch synthase family protein | Yes |

Note: Yes = expressed genes; No = lacking expression.

**Table S3.** Predicted genes within the candidate region based on the genomic sequence of Chinese Spring RefSeqv1.1. NBS-LRR (NLR) genes are marked in blue.

| Gene ID in CS | Location (bp) | Gene annotation |
|---|---|---|
| *TraesCS2B02G485600* | 2B:682848256-682860541 | ABC transporter-like |
| *TraesCS2B02G485700* | 2B:683001918-683005154 | Remorin, C-terminal |
| *TraesCS2B02G485800* | 2B:683021366-683029982 | SIT4 phosphatase-associated protein family |
| *TraesCS2B02G485900* | 2B:683030340-683030830 | RPM1-interacting protein 4/NOI4 |
| *TraesCS2B02G486000* | 2B:683034442-683040255 | RNA recognition motif domain |
| *TraesCS2B02G486100* | 2B:683042399-683049870 | **NBS-LRR disease resistance protein-like protein** |
| *TraesCS2B02G486200* | 2B:683053513-683062553 | **NBS-LRR disease resistance protein-like protein** |
| *TraesCS2B02G486300* | 2B:683065941-683075708 | **NBS-LRR disease resistance protein-like protein** |
| *TraesCS2B02G486400* | 2B:683127549-683135331 | **NBS-LRR disease resistance protein-like protein** |
| *TraesCS2B02G486700* | 2B:683159256-683166341 | **NBS-LRR disease resistance protein-like protein** |
| *TraesCS2B02G486800* | 2B:683170934-683172344 | — |
| *TraesCS2B02G486900* | 2B:683173679-683176334 | S-adenosyl-L-methionine-dependent methyltransferase |
| *TraesCS2B02G487000* | 2B:683307341-683309992 | Leucine-rich repeat domain superfamily, F-box-like domain superfamily |
| *TraesCS2B02G487100* | 2B:683483474-683487437 | Leucine-rich repeat domain superfamily, F-box-like domain superfamily |
| *TraesCS2B02G487200* | 2B:683728900-683729163 | ATP synthase, F0 complex, subunit b/b', bacterial/chloroplast |
| *TraesCS2B02G487300* | 2B:683731363-683731674 | 30S ribosomal protein S14, chloroplastic (RPS14) |
| *TraesCS2B02G487400* | 2B:683731820-683734978 | Photosystem I PsaA/PsaB |
| *TraesCS2B02G487500* | 2B:683737949-683739942 | Photosystem I assembly protein Ycf3 |
| *TraesCS2B02G487600* | 2B:683749450-683751731 | Rx, N-terminal |
| *TraesCS2B02G487700* | 2B:683752480-683755002 | **NBS-LRR disease resistance protein-like protein** |
| *TraesCS2B02G487800* | B:683758420-683760224 | B3 DNA binding domain, REM family |
| *TraesCS2B02G487900* | 2B:685146693-685148431 | Protein of unknown function DUF716 |
| *TraesCS2B02G488000* | 2B:685265721-685270904 | Zinc finger C2H2, NB-ARC, Leucine-rich repeat domain superfamily |
| *TraesCS2B02G488100* | 2B:685280054-685280419 | Protein of unknown function DUF3681 |
| *TraesCS2B02G488200* | 2B:685502873-685503238 | Protein of unknown function DUF3681 |
| *TraesCS2B02G488300* | 2B:685605585-685605950 | Protein of unknown function DUF3681 |
| *TraesCS2B02G488400* | 2B:685741193-685746862 | Zinc finger C2H2, NB-ARC, Leucine-rich repeat domain superfamily |
| *TraesCS2B02G488500* | 2B:686041063-686041428 | Protein of unknown function DUF3681 |
| *TraesCS2B02G488600* | 2B:686045632-686046972 | Zinc finger C2H2 superfamily; Rx, N-terminal |
| *TraesCS2B02G488700* | 2B:686049368-686050810 | Leucine-rich repeat domain superfamily |
| *TraesCS2B02G488800* | 2B:686055835-686056474 | — |
| *TraesCS2B02G489000* | 2B:686449733-686450098 | Protein of unknown function DUF3681 |
| *TraesCS2B02G489100* | 2B:686464719-686465090 | Protein of unknown function DUF3681 |
| *TraesCS2B02G489200* | 2B:686783473-686783685 | dUTPase-like superfamily |

| | | |
|---|---|---|
| *TraesCS2B02G489300* | 2B:686808260-686808625 | Protein of unknown function DUF3681 |
| *TraesCS2B02G489400* | 2B:686809725-686815511 | Zinc finger C2H2, AP2/ERF, NB-ARC, Leucine-rich repeat domain superfamily |
| *TraesCS2B02G489500* | 2B:686817703-686820112 | Bromodomain-like superfamily |
| *TraesCS2B02G489600* | 2B:686834230-686838629 | Protein kinase C-like, phorbol ester/diacylglycerol-binding domain |
| *TraesCS2B02G489700* | 2B:687205211-687207726 | Wall-associated receptor kinase |
| *TraesCS2B02G489800* | 2B:687227079-687228505 | Plant peroxidase |
| *TraesCS2B02G489900* | 2B:687248060-687248948 | FD-like 15 protein |
| *TraesCS2B02G490000* | 2B:687333892-687334206 | — |
| *TraesCS2B02G490100* | 2B:687468931-687474243 | Amine oxidase, FAD/NAD(P)-binding domain |
| *TraesCS2B02G490200* | 2B:687633963-687635975 | Chloramphenicol acetyltransferase-like domain superfamily |
| *TraesCS2B02G490300* | 2B:688060431-688061678 | F-box associated interaction domain |
| *TraesCS2B02G490400* | 2B:688234797-688235685 | Invertase/pectin methylesterase inhibitor domain superfamily |
| *TraesCS2B02G490500* | 2B:688239703-688243735 | Exonuclease, RNase T/DNA polymerase III |
| *TraesCS2B02G490600* | 2B:688375493-688377107 | Exonuclease, RNase T/DNA polymerase III |
| *TraesCS2B02G490700* | 2B:688430630-688434145 | FMN-dependent dehydrogenase |
| *TraesCS2B02G490800* | 2B:688456640-688459507 | Signal recognition particle, SRP14 subunit |
| *TraesCS2B02G490900* | 2B:688459906-688463027 | Aminomethyltransferase, folate-binding domain |
| *TraesCS2B02G491000* | 2B:688478196-688480660 | Tyrosinase copper-binding domain, Polyphenol oxidase, C-terminal |
| *TraesCS2B02G491100* | 2B:689142113-689144392 | Tyrosinase copper-binding domain, Polyphenol oxidase, C-terminal |
| *TraesCS2B02G491200* | 2B:689484272-689487239 | Autophagy-related protein |
| *TraesCS2B02G491300* | 2B:689713615-689715922 | Glycoside hydrolase, family 28; Parallel beta-helix repeat |
| *TraesCS2B02G491400* | 2B:689764554-689766778 | Ppo-B2 |
| *TraesCS2B02G491500* | 2B:689776118-689777712 | F-box domain, Leucine-rich repeat domain superfamily |
| *TraesCS2B02G491600* | 2B:689870686-689873964 | Protein of unknown function DUF594 |
| *TraesCS2B02G491700* | 2B:690026216-690034656 | Glycosyl transferase |

—, no conserved domains

**Table S4.** Twenty-two pairs of primers were developed from the seven expressed NLR genes and used to amplify the genomic DNAs of Kronos and Rusty. PCR products of Kronos and Rusty are presented in the last column (from left to right). All of the markers amplified the expected bands in Kronos. By contrast, only two of them amplified products in Rusty. The same DNAs were tested with primers *pku4856F2R2*, *pku4861F7R7*, *pku4886F3R3*, *pku4907F1R1* and *pku4917F3R3* (Table 2) to confirm that the lack of amplification in Rusty was not due to degraded DNA. K, Kronos; R, Rusty.

| Genes | Markers | Forward primer (5' - 3') | Reverse primer (5' - 3') | Ann.T (℃) | Expected size (bp)# | PCR products (Kronos Rusty) |
|---|---|---|---|---|---|---|
| *TRITD2Bv1G223210* | *TRI2B223210F1R1* | GTGGAGCCAAGACTTGATCGAT | CCGGAAGTGTACCTTGGAAGTAG | 60 | 5519 | 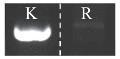 |
| | *TRI2B223210F2R2* | CTGTTTGGTTTTTCCGCTGTG | TGTAACGTGTTAGTGACGGTGATC | 60 | 5922 | 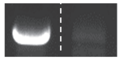 |
| | *TRI2B223210F3R3* | ACTGCTTATCCTTTATTCTCTTCT | GGTGAGCGTGTAAACTATGTC | 56 | 5716 | 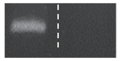 |
| | *TRI2B223210F7R7* | TGCCTTTTGGTTCACTAAAAC | ACCTGGCACATGAATTCTTCGT | 58 | 2309 | 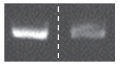 |
| *TRITD2Bv1G223370* | *TRI2B223370F1R1* | AGTATACTGATCACAGTGACAAAT | AACTCAGTGATATGCGGTGCG | 55 | 2035 | 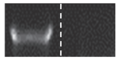 |
| | *TRI2B223370F2R2* | CTACATGATTTTAGGGTTCACAC | GCATTATCCAGAAGACACAGC | 56 | 2342 | 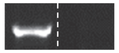 |
| | *TRI2B223370F3R3* | TCCATAGGATGTAGTTGGTTC | GTGTTATCTCAAAGTTGCTAGAAG | 55 | 3200 | 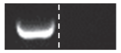 |
| | *TRI2B223370F4R4* | CATAGGATGTAGTTGGTTCTTG | ATCATTGGATCAGTGTCTGTGT | 58 | 3052 | 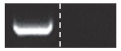 |
| *TRITD2Bv1G223450* | *TRI2B223450F1R1* | CCCTATTTTTCTTAGGTCA | AATGGTGGGTCAGTTATCAG | 52 | 1206 | 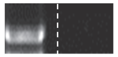 |
| | *TRI2B223450F2R2* | AATCTTTGGGTTGTGCTGTAG | ACGGGTGTTATCTGCGGTG | 54 | 1413 | 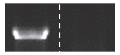 |
| | *TRI2B223450F3R3* | AAACCAAATGTAAGTACTGGAC | GAGTCAGTTGCTTAGCGAGT | 55 | 3817 | 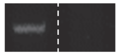 |

| | | | | | | |
|---|---|---|---|---|---|---|
| *TRITD2Bv1G223460* | *TRI2B223460F2R2* | TAAGCATAATCCCATTATATAACG | GAGCTACAGAAAACCACACTAGC | 57 | 2832 | 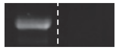 |
| | *TRI2B223460F3R3* | GCCATGTCGATACACTGAGTT | GCTCATACCTTCCACGGAAG | 57 | 3167 | 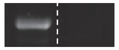 |
| *TRITD2Bv1G223490* | *TRI2B223490F1R1* | CTGTAATACGAAGACCAACGGTA | ATTTTCCATTCAATGCAATCGC | 56 | 2653 | 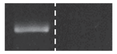 |
| | *TRI2B223490F2R2* | CAATGATCGTACTATGCCTGTTT | AATCTAATCCTAGGGTTCTAGTT | 58 | 2574 | 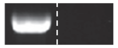 |
| | *TRI2B223490F3R3* | AAGGTAACAGTGCTGGCAAGTC | AGATTGCCTCAAAAGCAACGC | 62 | 4639 | 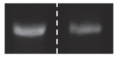 |
| *TRITD2Bv1G223550* | *TRI2B223550F1R1* | GCATCGCTAGTCCATGTCG | GAGCACCTTCAGTTCTCAAT | 58 | 789 | 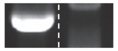 |
| | *TRI2B223550F2R2* | CGTTTGATCGGACGATACG | TCAATGCTCAAGCCAACTAAG | 60 | 680 | 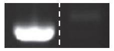 |
| | *TRI2B223550F6R6* | CCACTTTCTTCTTCTGTTCGCATT | GGACCATCTATAGTTGCACACTCA | 58 | 4018 | 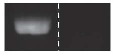 |
| *TRITD2Bv1G223640* | *TRI2B223640F1R1* | TTGGCACAGCTACAAGGGGA | AATGCGTGAGGAATAAGAGTTAGG | 60 | 5110 | 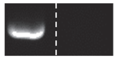 |
| | *TRI2B223640F2R2* | ACAGCTACAAGGGGAGAGACA | TGCGTGAGGAATAAGAGTTAGG | 59 | 5103 | 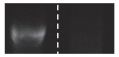 |
| | *TRI2B223640F3R3* | ATCTCAATCTGATCAAGGACG | ACTTTTGGATAAACTGAAAGGC | 58 | 5169 | 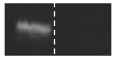 |
| *TRITD2Bv1G223060* | *pku4856F2R2* | TCCTTGGTCATCGAGATAGG | GCTGGTCAAAGCTTGAATTTG | 52 | 390 | 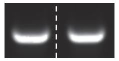 |
| *TRITD2Bv1G223210* | *pku4861F7R7* | CTTTGGGGGTAATAGACACTCTA | TGATTCCCACCCTGTTCTTG | 54 | 429 | 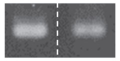 |
| *TRITD2Bv1G223450* | *pku4886F3R3* | CCAACTGTGCTGGTTCCTT | TTGCTTTGATTGGCTGTCTAA | 52 | 640/712 | 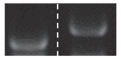 |
| *TRITD2Bv1G224020* | *pku4907F1R1* | TTCCAGCTTTATGTACGTGTAGT | TCCATTCAGGACGAAGTGC | 58 | 671 | 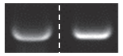 |
| *TRITD2Bv1G224370* | *pku4917F3R3* | TCAATAGGCTGAGATAACTGC | TGTGTACCCAAAGAAGAAGG | 52 | 1400 | 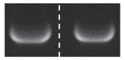 |

**Table S5.** Detection of *SrKN* resistance based on the haplotype of five linked markers (*pku4856F2R2*, *pku4861F7R7*, *pku4886F3R3*, *pku4907F1R1* and *pku4917F3R3*). A collection of 23 accessions of *T. turgidum* ssp. *durum* and 16 of *T. aestivum* was tested. All these lines were evaluated with *Pgt* race 34MKGQM. Gene postulation was based upon infection types and genotypes from the diagnostic marker of *Sr13*. A, T4-3102 allele; B, Rusty allele; -, susceptible or with unknown gene(s).

| Cultivars/Lines | Species | Markers | | | | | Race | Gene Postulation |
|---|---|---|---|---|---|---|---|---|
| | | ***pku4856F2R2*** | ***pku4861F7R7*** | ***pku4886F3R3*** | ***pku4907F1R1*** | ***pku4917F3R3*** | **34MKGQM** | |
| T4-3102 (*SrKN*) | *T. durum* | A | A | A | A | A | 1+ | *SrKN* |
| Vernal (*Sr9e*) | *T. durum* | A | A | A | A | A | 1+ | *Sr9e* |
| Rusty | *T. durum* | B | B | B | B | B | 4 | - |
| Varna AC03-1002478 | *T. durum* | B | B | B | B | B | 3+ | - |
| Bekaa AC03-1002490 | *T. durum* | B | B | B | B | B | 3 | - |
| Candeal AC03-1002491 | *T. durum* | B | B | B | B | B | 4 | - |
| Stewart 63 AC03-1002492 | *T. durum* | B | B | B | B | B | 4 | - |
| Minas NO251 AC03-1002495 | *T. durum* | B | B | B | B | B | 4 | - |
| Kamilaroi AC03-1002497 | *T. durum* | B | B | B | B | B | 4 | - |
| Odin AC03-1002499 | *T. durum* | B | B | B | B | B | 3+ | - |
| Pseudo-Compressom Va AC03-1002503 | *T. durum* | B | B | B | B | B | 3- | - |
| Lal Batala AC03-1002504 | *T. durum* | B | B | B | B | B | 4 | - |
| Malta AC03-1002505 | *T. durum* | B | B | B | B | B | 3 | - |
| Crete 1 AC03-1002512 | *T. durum* | B | B | B | B | B | 3 | - |
| Svevo | *T. durum* | A | A | A | A | A | 1+ | *SrKN* or *Sr9e* |
| Langdon | *T. durum* | A | A | A | A | A | ; / 1- | *SrKN* or *Sr9e* and *Sr13* |
| PI 94701 | *T. durum* | B | B | B | B | B | 2+ | *Srdp2* |

| | | | | | | | | |
|---|---|---|---|---|---|---|---|---|
| Nanking AC03-1002498 | *T. durum* | A | A | no PCR Pro. | no PCR Pro. | no PCR Pro. | 0 | *Sr13* |
| AUS# 15878 AC03-1002506 | *T. durum* | B | A | B | no PCR Pro. | A | ; | *Unknown* |
| Akathiotiko AC03-1002513 | *T. durum* | B | B | B | B | B | ; | *Sr13* |
| Acme AC03-1002514 | *T. durum* | B | B | B | B | B | ; | *Unknown* |
| Candeal AC03-1002489 | *T. durum* | A | B | B | B | B | 1 | *Sr13* |
| Kenya 112 AC03-1002516 | *T. durum* | B | B | B | B | B | ; | *Sr13* |
| Vernstein (*Sr9e*) | *T. aestivum* | A | A | A | A | A | 1+ / 2- | *Sr9e* |
| W2691*Sr9b* | *T. aestivum* | A | A | no PCR Pro. | no PCR Pro. | A | 4 | *Sr9b* |
| Cn*Sr9g* | *T. aestivum* | A | A | A | A | A | 4 | *Sr9g* |
| ISr9d-Ra | *T. aestivum* | A | B | no PCR Pro. | B | H | 4 | *Sr9d* |
| ISr9a-Ra | *T. aestivum* | A | A | A | A | A | 4 | *Sr9a* |
| ISr8a-Ra | *T. aestivum* | B | B | B | B | B | 3 | *Sr8a* |
| ISr5-Ra | *T. aestivum* | B | B | B | B | B | 4 | *Sr5* |
| Jinmai90 | *T. aestivum* | A | B | B | B | B | 4 | - |
| Yannong23 | *T. aestivum* | A | B | B | B | B | 4 | - |
| Jimai1 | *T. aestivum* | B | B | B | B | B | 4 | - |
| Jimai19 | *T. aestivum* | A | B | B | B | B | 4 | - |
| Lumai21 | *T. aestivum* | B | B | B | B | B | 4 | - |
| Jimai21 | *T. aestivum* | B | B | no PCR Pro. | B | B | 4 | - |
| Nanda2419 | *T. aestivum* | A | B | B | B | B | 3 | - |
| Ningchun4 | *T. aestivum* | B | B | B | B | A | 4 | - |
| Jinan17 | *T. aestivum* | A | B | B | B | B | 4 | - |

Note, no PCR Pro. = no PCR products.

## References


Li, T., Wu, X., Xu, X., Wang, W., and Cao, Y. (2016). Postulation of seedling stem rust resistance genes of Yunnan wheat cultivars in China. *Plant Protect Sci* 52(4), 242-249.

Li, T.Y., Ma, Y.C., Wu, X.X., Chen, S., Xu, X.F., Wang, H., et al. (2018). Race and virulence characterization of *Puccinia graminis* f. sp. *tritici* in China. *PloS One* 13(5), e0197579.

Chao, S., Rouse, M.N., Acevedo, M., et al. Evaluation of genetic diversity and host resistance to stem rust in USDA NSGC durum wheat accessions. The plant genome, 2017, 10(2), plantgenome2016.07.0071. https://doi.org/10.3835/plantgenome2016.07.0071.

Chen, S., Rouse, M.N., Zhang, W., Zhang, X., Guo, Y., Briggs, J., et al. (2020). Wheat gene *Sr60* encodes a protein with two putative kinase domains that confers resistance to stem rust. *New Phytol* 225(2), 948-959.

Saini, J., Faris, J.D., Zhang, Q., Rouse, M.N., Jin, Y., Long, Y., et al. (2018). Identification, mapping, and marker development of stem rust resistance genes in durum wheat 'Lebsock'. *Mol Breeding* 38(6), 1-14.
